# Unique lipid composition maintained by extracellular blockade leads to prooncogenicity

**DOI:** 10.1038/s41420-024-01971-y

**Published:** 2024-05-08

**Authors:** Kai Kudo, Ryo Yanagiya, Masanori Hasegawa, Joaquim Carreras, Yoshimi Miki, Shunya Nakayama, Etsuko Nagashima, Yuji Miyatake, Kan Torii, Kiyoshi Ando, Naoya Nakamura, Akira Miyajima, Makoto Murakami, Ai Kotani

**Affiliations:** 1 Department of Innovative Medical Science, Tokai University School of Medicine, Isehara, Kanagawa Japan; 2 Division of Hematological Malignancy, Institute of Medical Sciences, Tokai University, Isehara, Kanagawa Japan; 3 Department of Hematology and Oncology, Tokai University School of Medicine, Isehara, Kanagawa Japan; 4 Laboratory of Regulation of Infectious Cancer, Division of Cellular and Molecular Biology, Research Institute for Microbial Diseases, Osaka University, Suita, Osaka Japan; 5 Department of Urology, Tokai University School of Medicine, Isehara, Kanagawa Japan; 6 Department of Pathology, Tokai University School of Medicine, Isehara, Kanagawa Japan; 7 Laboratory of Microenvironmental Metabolic Health Sciences, Center for Disease Biology and Integrative Medicine, Graduate School of Medicine, The University of Tokyo, Tokyo, Japan; 8 Laboratory of Veterinary Physiology, College of Bioresource Science, Nihon University, Fujisawa, Kanagawa Japan

**Keywords:** Cancer, Diseases, Cancer metabolism

## Abstract

Lipid-mediated inflammation is involved in the development and malignancy of cancer. We previously demonstrated the existence of a novel oncogenic mechanism utilizing membrane lipids of extracellular vesicles in Epstein–Barr virus (EBV)-positive lymphomas and found that the lipid composition of lymphoma cells is skewed toward ω-3 fatty acids, which are anti-inflammatory lipids, suggesting an alteration in systemic lipid composition. The results showed that arachidonic acid (AA), an inflammatory lipid, was significantly reduced in the infected cells but detected at high levels in the sera of EBV-positive patients lead to the finding of the blockade of extracellular AA influx by downregulating FATP2, a long-chain fatty acid transporter that mainly transports AA in EBV-infected lymphoma cells. Low AA levels in tumor cells induced by downregulation of FATP2 expression confer resistance to ferroptosis and support tumor growth. TCGA data analysis and xenograft models have demonstrated that the axis plays a critical role in several types of cancers, especially poor prognostic cancers, such as glioblastoma and melanoma. Overall, our in vitro, in vivo, in silico, and clinical data suggest that several cancers exert oncogenic activity by maintaining their special lipid composition via extracellular blockade.

## Introduction

Homeostasis is maintained through the regulation of intra- and extra-cellular lipid flows to keep an adequate balance. In malignant tumors, this balance is often disrupted to create a composition favorable for tumor cells [1–4]. It is well known that the alteration of lipid composition by tumors induces various biological effects via the production of lipid mediators [5, 6]. Recently, we found that in Epstein–Barr virus (EBV)-positive lymphomas, a type of diffuse large B-cell lymphoma (DLBCL), the amount of polyunsaturated fatty acids (PUFAs) is markedly increased in tumor cells compared to that in normal B cells [7, 8]. This lipid composition extends to phospholipids in extracellular vesicles secreted from tumor cells, exerting biological effects in the extracellular environment. However, the mechanism and biological significance of changes in the lipid composition of cells caused by EBV infection or transformation remain elusive.

In recent years, “ferroptosis”, cell death caused by membrane lipid peroxidation, has attracted attention as an important biological event triggered by changes in cellular lipid composition [9–12]. During ferroptosis, cell membrane damage caused by the peroxidation of phospholipid-bound PUFA occurs in an iron ion-dependent manner, leading to caspase-independent cell death [13]. Therefore, a higher content of PUFAs, which are more easily oxidized, leads to higher sensitivity to ferroptosis in cells. For example, it has been known that the transformation of ferroptosis-resistant epithelial cancer cells into mesenchymal cancer cells causes the desaturation of membranous phospholipids which makes the cells ferroptosis sensitive [14]. In contrast, exogenous monounsaturated fatty acids (MUFAs) acyl-CoA synthase 3 (ACSL3) antagonizes PUFAs, which specifically blocks the accumulation of lipid reactive oxygen species in cell membranes and inhibits ferroptosis [15]. Canonically, ferroptosis is caused by the failure of glutathione peroxidase 4 (GPX4), which reduces the function of peroxided fatty acids in phospholipids of cell membranes. Magtanong et al. have recently proved that the sensitivity of ferroptosis can be regulated by the fatty acid composition of the phospholipids in the membrane even without GPX4 [15], suggesting that the unique lipid composition of the cell, so-called “lipoquality” (quality of lipids, as termed in modern lipid biology) is involved in the determination of the sensitivity of ferroptosis. Fatty acid desaturases, such as *FADS1*, *FADS2*, and *SCD1* are known to play major roles in changes in lipid composition by catalyzing fatty acid desaturation [16–18]. However, since these desaturases in mammals cannot make ω-3 and ω-6 fatty acids, which can serve as direct precursors of lipid mediators, the acquisition of these PUFAs in each cell is largely dependent on their influx from extracellular sources [19]. Recently, it was reported that the influx of extracellular fatty acids through CD36 into CD8^+^ T cells, the main players in anti-tumor immunity, induces ferroptosis and reduces anti-tumor immune functions [20]. Although many studies have reported the sensitivity of tumor cells to ferroptosis [21–24], their resistance has been poorly documented, and the universal mechanism for the acquisition of resistance throughout various histopathological types remains largely unknown.

In this study, we demonstrated that EBV-positive lymphoma cells were resistant to ferroptosis by blocking extracellular arachidonic acid (AA) influx via downregulation of fatty acid transport protein 2 (FATP2; encoded in *SLC27A2*). Furthermore, this mechanism plays an important role in the acquisition of ferroptosis resistance in some types of tumors. We believe that these findings may provide novel insights into survival strategies via the regulation of AA influx in various types of tumor cells, including EBV-positive lymphomas.

## Results

### AA in the serum and EBV-infected tumor cells exhibited opposite composition

**Fig. 1:**
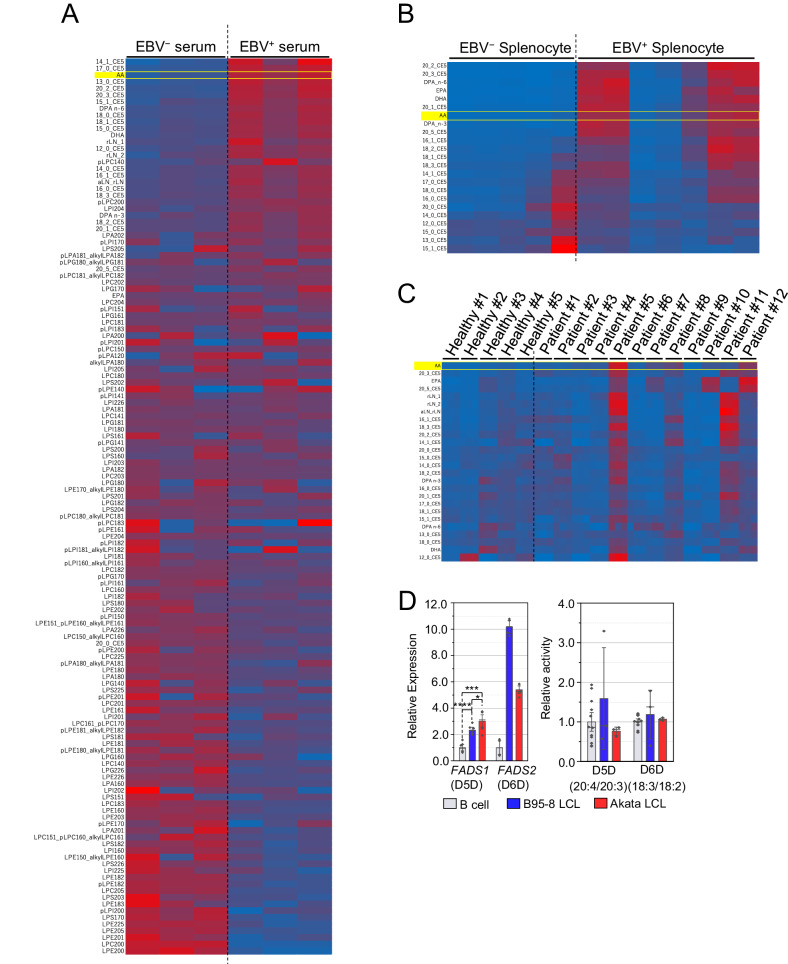
EBV-related disorders show accumulation of AA in serum. Fatty acid composition of serum collections (**A**) or splenocytes (**B**) derived from human hematopoietically humanized mice (*n* = 3), and those with EBV (Akata-strain) infection (*n* = 3). **C** Fatty acid composition of human serum derived from healthy volunteers (*n* = 5), and patients of EBV-associated disorders (*n* = 12). All samples were independently measured three times. **D** mRNA expressions of *FADS1* and *FADS2* (left), and intracellular activity of D5D and D6D (right) in B cell derived from healthy donor (gray), B95-8 LCL (blue), and Akata LCL (red).

Previously, we reported that the composition of the AA in the phospholipids was significantly reduced by EBV infection [8]. In order to elucidate the mechanism of the results, we measured the free AA in the serum and tumor tissue in the EBV-infected mice. The EBV-infected mice had higher levels of free AA in the serum but a slightly lower level of free AA in the spleen than the non-infected mice (Fig. 1A, B). Free AA levels in the sera of several patients with EBV-lymphoproliferative disease (LPD) were higher than in healthy controls (Fig. 1C). Both expression and metabolic activity of fatty acid desaturase 1 (FADS1) and FADS2 which synthesized AA from the precursor was not altered (Fig. 1D), suggesting that PUFA synthesis is unlikely to be involved in AA reduction in EBV-infected large cell lymphomas (LCLs). Based on the results, we hypothesized that EBV infection reduced the influx of free AA into the tumor cells.

### AA influx mediated by FATP2 regulates ferroptosis in Akata LCLs

**Fig. 2:**
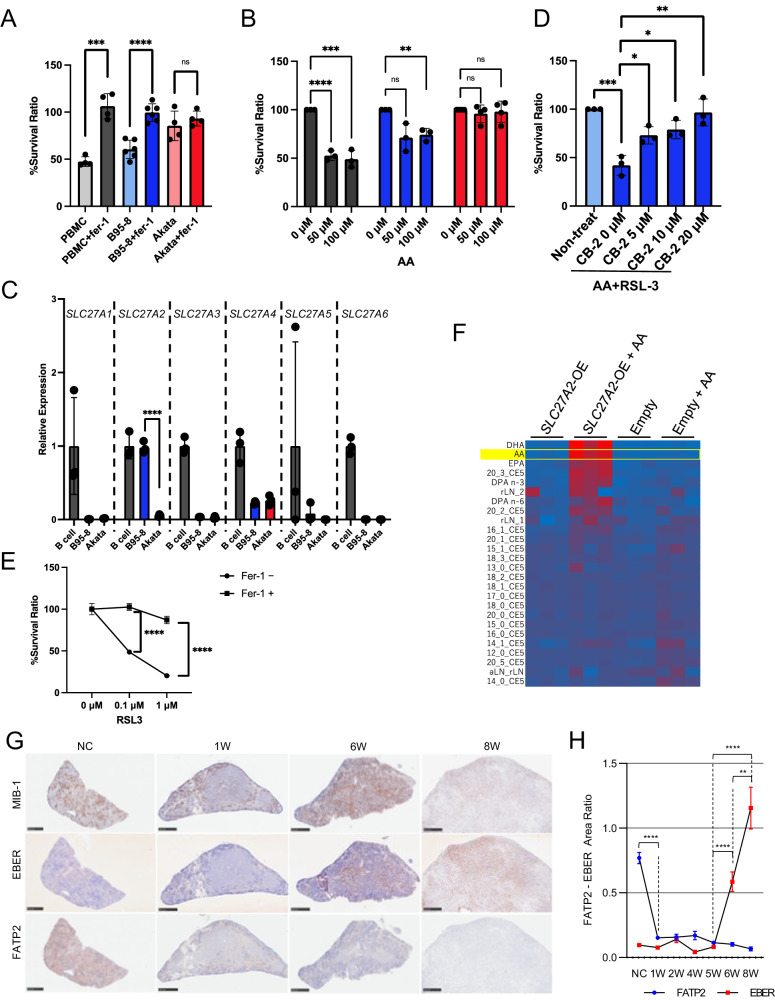
Blockade of serum AA influx due to *SLC27A2* downregulation results in resistance against ferroptosis in Akata LCLs. **A** PBMCs derived from healthy volunteers (*n* = 4), B95-8 LCLs (*n* = 6), and Akata LCLs (*n* = 4) were treated with 1 μM RSL-3 (a ferroptosis inducer) with or without 2 μM Fer-1 (a ferroptosis inhibitor), and each cell viability was detected as fluorescence intensity in Alamar blue assay and calculated relative to the value in RSL-3(−) cells. **B** Peripheral B cells derived from healthy volunteers (*n* = 3; gray), B95-8 LCLs (*n* = 3, blue), and Akata LCLs (*n* = 4, Red) were treated with 0.1 µM of RSL-3 and 0, 50, and 100 µM of AA. Each cell viability was detected as fluorescence intensity in Alamar blue assay and calculated relative to the value in AA-free group. **C** Relative expression *SLC27A* family mRNA in B95-8 LCLs (*n* = 3 in each) and Akata LCLs (*n* = 3 in each), relative to those in peripheral B cells derived from healthy volunteers (*n* = 3). **D** B95-8 LCLs (*n* = 3) were treated with CB-2 (a FATP2 inhibitor) at the indicated concentrations accompanied with 0.1 μM of RSL-3 and 100 μM of AA after 3 h of pre-incubation. Each cell viability was detected as fluorescence intensity in Alamar blue assay and calculated relative to the value in CB-2-free group. **E** *SLC27A2*-overexpressed Akata LCLs were treated with 0, 0.1, and 1 µM of RSL-3 and 100 µM of AA with or without 2 µM of Ferrostatin-1 (*n* = 3 in each). Survival ratio was calculated relative to the value of RSL-3-free group. **F** *SLC27A2*-overexpressed Akata LCLs (*SLC27A2*-OE) and empty vector-transfected Akata LCLs (Empty) were incubated with or without 100μM of AA for an hour and then cellular fatty acid compositions were measured (*n* = 3 in each). Representative in situ hybridization of EBER and immunohistochemical staining of MIB-1 (Ki-67) and FATP2 in the spleen of EBV-infected mice. Tissues were collected at the indicated periods after EBV infection (**G**). Scale bar, 500 nm. The ratios of positive area /total cells for EBER and FATP2 were measured (**H**).

In order to investigate the biological significance of reduced AA in EBV-infected cells, ferroptosis was induced by its inducer RSL-3 with or without its inhibitor ferrostatin-1 (Fer-1) (Fig. 2A) in EBV-transformed cells (B95-8 LCL and Akata LCL) as well as the healthy human-derived peripheral blood monocyte (PBMC). Considerable cell death by RSL-3, followed by its inhibition by Fer-1, was observed in B95-8 LCLs and PBMCs, whereas Akata LCLs were barely affected by RSL-3 or Fer-1, indicating strong resistance to ferroptosis. These results were confirmed by morphological changes to smaller sizes and lipid peroxidation upon the induction of ferroptosis (Supplementary Fig. 1A, B).

Akata LCLs were resistant to ferroptosis even after AA supplementation (Fig. 2B) which again suggests that the AA influx in Akata-infected cells was blocked. The FATP family is involved in the uptake of long-chain unsaturated fatty acids. The *SLC27A2*, which encodes FATP2, is a representative transporter of AA, was downregulated in Akata LCLs, but not in B95-8 LCLs nor primary B cells (Fig. 2C). This finding was compatible with ferroptosis sensitivity. The pharmacological inhibition of AA influx using CB-2, a selective inhibitor of FATP2, reduced the cellular levels of AA and AA-derived oxidized fatty acids (lipid mediators) (Supplementary Fig. 1C, D) and conferred resistance to ferroptosis in B95-8 cells in a dose-dependent manner (Fig. 2D). In addition, the overexpression of *SLC27A2* in Akata LCLs (Supplementary Fig. 1E), which showed improvement of sensitivity to ferroptosis (Fig. 2E), demonstrated significantly higher AA in phospholipids upon the addition of AA (Fig. 2F). The results were reproduced by humanized EBV-infected LPDs mice. While the FATP2 expression in the spleen was prominently detected in non-infected mice, that in the humanized EBV-infected LPDs mice rapidly reduced only 1 week after viral infection resulting in the negativity at the tumor development (Fig. 2G, H). Those results suggest that downregulation of *SLC27A2* in Akata LCLs causes resistance to ferroptosis.

### *SLC27A2* overexpression negatively regulates in vivo tumor growth of Akata LCLs

**Fig. 3:**
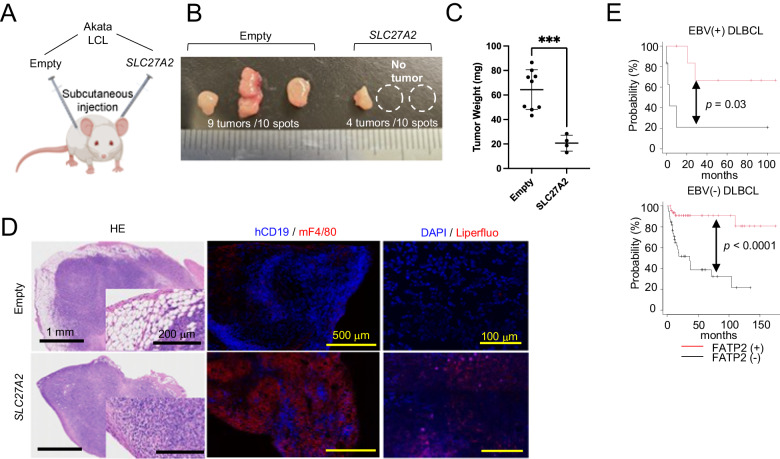
*SLC27A2* overexpression negatively regulates in vivo tumor growth of Akata LCLs. **A** Development of a xenograft tumor model in NOG mice. *SLC27A2-*overexpressed Akata LCLs (*SLC27A2)* and control Akata LCLs (Empty) were injected subcutaneously into the left and right shoulder of the same individual (1 × 10^6^ cells/50 µl/injection). Tumors were resected 5 weeks after injection (**B**), then measured tumor weight (**C**). **D** Microscopic appearance of injected tumors. Immunohistochemically (central), human CD19 (red), and mouse F4/80 (blue) were stained with fluorescent-conjugated antibodies. In Liperfluo staining (right), the red and blue signals indicate the presence of lipid peroxides and nuclei (DAPI), respectively. **E** Survival analyses of DLBCL patients: FATP2(+) (*n* = 7) and FATP2(−) (*n* = 6) cases in EBV-positive DLBCL and FATP2(+) (*n* = 47) and FATP2(−) (*n* = 39) cases in EBV-negative DLBCL.

Next, to examine whether the overexpression of *SLC27A2* affects tumor growth and tissue peroxidation in vivo, subcutaneous tumors by injecting *SLC27A2*-overexpressed Akata LCLs (*SLC27A2*) or empty vector-transfected Akata LCLs (Empty) into the left and right shoulders of non-obese diabetic (NOD)/Shi-SCID, interleukin-2 receptor-null mice (NOG mice) (Fig. 3A). After 5 weeks, the viability and size of the *SLC27A2* tumor tissues were significantly lower than those of the empty tissues (Fig. 3B, C). Morphologically, the control tumor tissues showed a significant accumulation of lipids just below the capsule, where many tumor cells had infiltrated, whereas *SLC27A2* tumor tissues were filled with F4/80-positive inflammatory cells and only a few, if any, tumor cells. Accumulation of lipid peroxides, as assessed by Liperfluo (Fig. 3D), a pattern similar to that of the subcutaneous tumor tissue with B95-8 cells (Supplementary Fig. 2A), was observed in *SLC27A2* tumor tissue. Lipidomic analysis of these tumor tissues showed a significant increase in the free AA ratio (Supplementary Fig. 2B) as well as in arachidonoyl-phosphatidylcholine (PC) and -phosphatidylethanolamine (PE) per tumor cell (Supplementary Fig. 2C, D) in *SLC27A2* mice. We calculated the AA/18:1 ratio in the phospholipids of tumor tissues, since oleic acid (18:1) has been recently reported to have an inhibitory effect on ferroptosis induction [15, 25], which showed that the ratio was higher in *SLC27A2* tumors (Supplementary Fig. 2E). Various metabolites derived from AA were upregulated in *SLC27A2* tumors (Supplementary Fig. 2F). Altogether, these results suggest that the overexpression of *SLC27A2* suppress tumor growth by the accumulation of lipid peroxides. In addition to that, the overproduction of AA-derived lipid mediators could recruit inflammatory cells and establish a disadvantageous microenvironment for tumor growth. We further investigated the relationship between FATP2 expression in immunohistochemistry and clinical outcomes of DLBCL patients, which is the most common subtypes of B-cell lymphoma. FATP2 expression was related to better overall survival in both EBV-positive and EBV-negative DLBCL cases (Fig. 3E) which suggests that the results obtained in the mouse model and in vitro experiments were recaptilated in human DLBCL.

### *SLC27A2*^low^ tumors have a poor prognosis in several types of malignant tumors

**Fig. 4:**
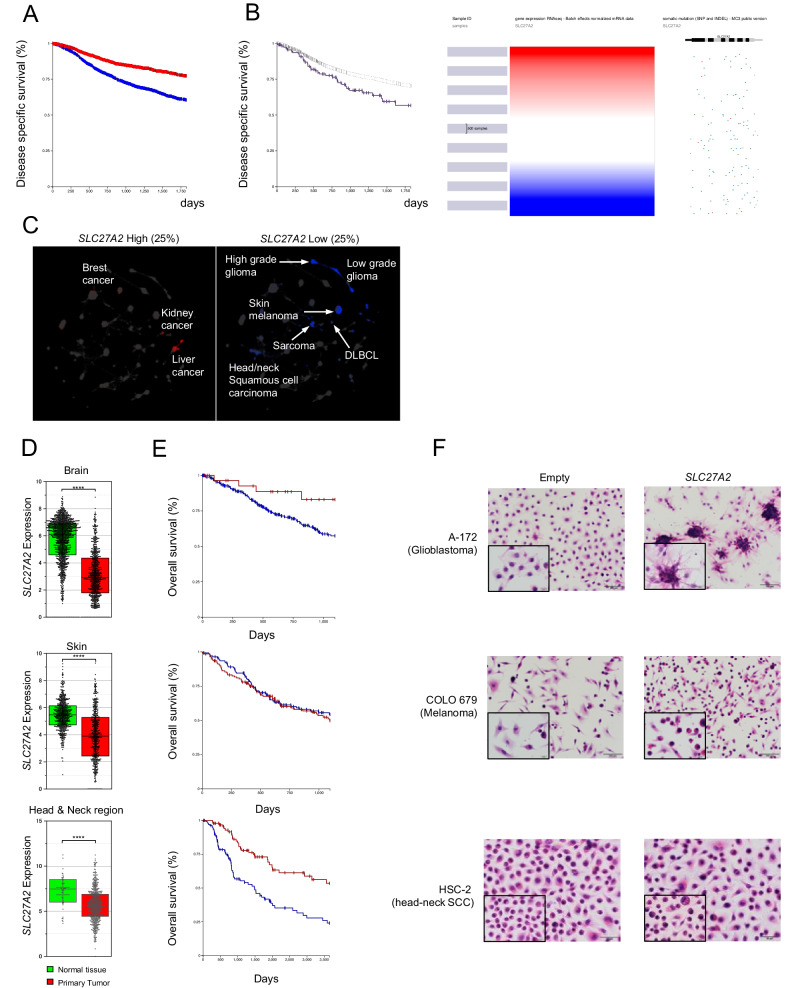
The impact of downregulation of FATP2 in human cancers. **A** Impact of *SLC27A2* expression on prognosis in all cancers extracted from the TCGA database. Survival curves were generated using the top 25% (2318 cases) and bottom 25% (2327 cases) of *SLC27A2* expression within the 9330 total extracted cases. Analysis was performed on UCSC Xena. **B** Impact of mutation with *SLC27A2* expression on prognosis in all cancers extracted from the TCGA database. Survival curves were generated using the cases which have a mutation (117 cases) and no mutation (8670 cases) in *SLC27A2*. Analysis was performed on UCSC Xena. **C** Tumor map projection of all cancer patient data extracted from the TCGA database; only the top 25% and bottom 25% of *SLC27A2* expression cases were extracted and projected onto the map. **D** Comparison of *SLC27A2* expression levels in cancer patients and normal tissue at each of the three *SLC27A2*^low^ histologic types (glioblastoma, melanoma, and HNSQ). **E** Relationship between *SLC27A2* expression and prognosis in cancer cases extracted in (**D**). Analysis was performed on UCSC Xena. **F** Control vector-transfected cells (Empty) and *SLC27A2*-overexpressed cells (*SLC27A2*) were stained with hematoxylin and eosin. Values in (**D**) are presented as mean ± SEM. Student’s *t*-test: ****p < 0.0001.

To investigate whether the *SLC27A2*–AA–ferroptosis axis can be applied to other cancers, in silico analysis was performed using a dataset of cancer patients registered in The Cancer Genome Atlas (TCGA). Disease-specific survival analysis of the top 25% and bottom 25% of *SLC27A2* expression among 10,000 cases revealed that *SLC27A2*^low^ tumors had a significantly poorer prognosis (Fig. 4A and Supplementary Fig. 3). The correlation between the presence or absence of *SLC27A2* loss-of-function mutations and prognosis in the same group of cases showed that the group with the mutation had worse prognosis (Fig. 4B). When the datasets were projected onto a tumor map to visualize individual tumor types with high and low *SLC27A2* expression, *SLC27A2*^low^ cases were clustered into generally poor prognosis cancer types, such as high-grade gliomas (glioblastomas) and cutaneous melanomas, head and neck region squamous cell carcinoma (HNSQ), and sarcomas (Fig. 4C). *SLC27A2* expression was significantly lower in primary tumors than in normal tissues in glioblastomas, cutaneous melanomas, and HNSQ (Fig. 4D). In glioblastomas and melanomas*, SLC27A2*^low^ cases had a poorer prognosis than *SLC27A2*^high^ cases, whereas there was no significant difference between the high- and low-expression groups in the HNSQ (Fig. 4E). Moreover, *SLC27A2* was ectopically expressed in *SLC27A2*^low^ tumor cell lines, which were predicted to be poor prognostic by in silico analysis. Marked morphological changes were observed in glioblastoma (A-172) and melanoma (COLO679) cells, but not in HNSQ (HSC-2) cells following ectopic *SLC27A2* expression (Fig. 4F). These results suggest that the *SLC27A2*–AA–ferroptosis axis is involved in at least several cancers.

### Ectopic *SCL27A2* expression inhibits tumor growth

**Fig. 5:**
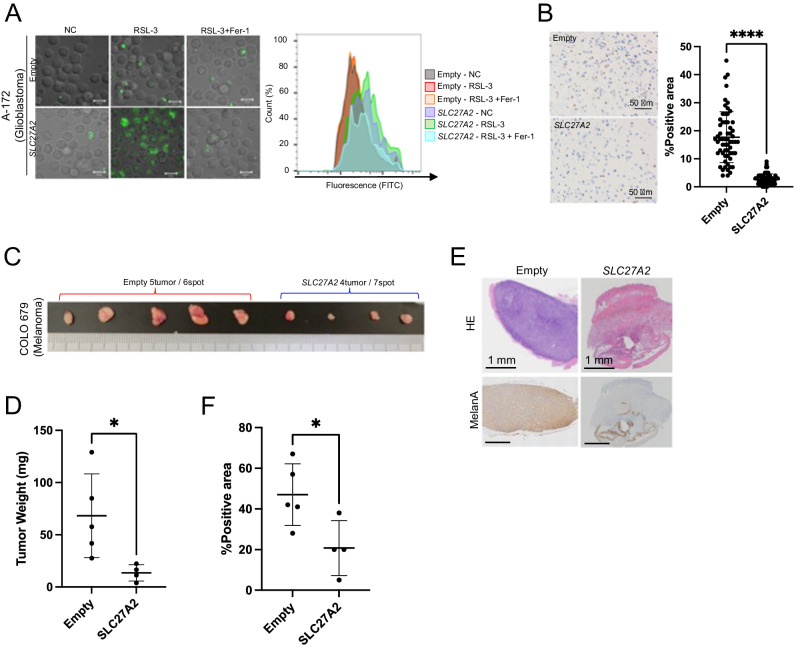
Anti-tumor effects mediated by AA influx and ferroptosis in *SLC27A2*^low^ tumors. **A** Ferroptosis induction and Liperfluo staining in *SLC27A2*-overexpressed A172 cells. RSL-3 and fer-1 were added at 0.1 and 2 μM, respectively. **B** Representative Ki-67 immunostaining of A172 cell administered brain tissue. The percentages of Ki-67-positive and negative cells were calculated by observation of 20 high-power fields of view in the administrated hemisphere. **C** Macrograph of subcutaneous tumor tissue with COLO679 cells. Tumor tissue was resected 28 days after tumor cell administration. **D** The weight of COLO679 subcutaneous tumor tissue (*n* = 4). **E** Representative micrographs of COLO679 subcutaneous tumor tissue. The upper panel shows the HE-stained image and the lower panel shows the MelanA immunostained images. **F** MelanA positivity in COLO679 subcutaneous tumor tissue. MelanA-positive regions were extracted from the whole area of tumor tissue and quantified by ImageJ (*n* = 4). Values in (**B**, **D**, **F**) represent the mean ± SD. Student’s *t*-test: *p < 0.05, ****p < 0.0001.

The lipid peroxidation signal increased in A-172 cells with ectopic expression of *SLC27A2* (*SLC27A2*-A-172) at ferroptosis (Fig. 5A). When A-172 cells with or without ectopic *SLC27A2* expression were administered intracranially, the number of viable tumor cells (human Ki-67 positive cells) was significantly reduced in mice with *SLC27A2*-A-172 cells compared to that in control cells (Fig. 5B). These results suggested that glioblastoma development depends on the SLC27A2–AA–ferroptosis axis, as in the case of Akata LCLs. In the subcutaneous tumors generated by COLO679 engraftment, tumor size and positivity for MelanA, a specific marker of melanoma, were significantly lower in the *SLC27A2*-overexpressing group (*SLC27A2*-COLO679) than in the control group (Fig. 5C–F).

These results suggest that the *SLC27A2*–AA–ferroptosis axis may contribute to several cancers, such as EBV-negative lymphomas, glioblastoma, melanoma, and possibly other tumors.

### BART miRNAs are involved in hypoxia phenotypes

*SLC27A2* has been reported to be downregulated by hypoxia, and its signature was enriched in *BamHI A rightward transcript* (*BART*) miRNA-overexpressing THP-1 (*BART*/THP-1) (Supplementary Fig. 4A). Expression of *BART* miRNAs is one of the major differences between the Akata LCLs (*BART* miRNA^+^) and B95-8 LCLs (*BART* miRNA^−^) [26]. Additionally, incubation of THP-1 and HL-60, a leukemic cell line, under hypoxic conditions (1% O_2_, 6 h, 37 °C) resulted in decreased expression of *SLC27A2* in both cell lines (Supplementary Fig. 4B). These results suggest that the *BART* miRNA-hypoxia axis may be responsible for the downregulation of *SLC27A2* in Akata LCLs.

## Discussion

In this study, we demonstrated the contradictory composition of free PUFA between serum and cellular AA in EBV-related LPDs. This discrepancy was caused by the downregulation of FATP2, and limited AA inflow resulted in the resistance to ferroptosis. In addition, the FATP2–AA–ferroptosis axis was also suggested to be one of the important mechanisms in other cancers, such as glioblastoma and melanoma.

FATP2 expression in patients with lymphoma did not differ significantly between EBV-positive and EBV-negative lymphomas. Although further investigations using a large number of EBV-positive lymphomas are needed, one possible reason for the small difference might be the deletion of the genome encoding BART miRNA found in a considerable number of EBV-positive malignant lymphoma cases, which is partly responsible for the downregulation of FATP2 [27]. Still, FATP2-negative DLBCL patients, regardless of EBV (+) or (−) cases, had a worse prognosis. These results suggest that regardless of EBV infection, FATP2 has some anti-tumorigenic actions, likely by promoting ferroptosis in lymphoid malignancies.

Although FATP2 in immune cells may be important for anti-tumor immunity via the production of AA-derived lipid mediators, decreased FATP2 expression in tumor cells may contribute to the evasion of immune surveillance by suppressing the production of such pro-inflammatory mediators, potentially leading to increased malignancy. In contrast, the blockade of FATP2 has recently been shown to exert mainly anti-tumorigenic function [28–32]. In thyroid cancer and neuroblastoma, the blockade of FATP2 activity suppresses tumor growth. Our in silico analysis showed that these tumors express high levels of FATP2 and that their expression has no impact on prognosis (data not shown). In melanoma, the function of FATP2 appears complicated; FATP2 is pro-tumorigenic in melanoma of the elderly, whereas it is not expressed in melanoma of the young [31]. Our analysis of the TGCA database included all melanoma cases, including primary and metastatic melanomas, and showed that FATP2^low^ patients displayed a worse prognosis. The number of patients with metastatic melanomas, with a median age of 50’s, was much greater than that of patients with primary melanomas, with a median age of about 70’s, suggesting that at least aged melanoma was a minority in the dataset. In these cancers, the acyl-CoA synthetase activity of FATP2 was proposed to be critical for its pro-tumorigenic activity, whereas, in EBV-infected LCLs, the transport of AA via FATP2 alone was sufficient for the induction of ferroptosis. Thus, the function of FATP2 may differ depending on the type or context of the cancer. Recent studies have shown that CD36, a pan-lipid transporter [33, 34], causes ferroptosis in CD8+ T cells rather than in tumor cells in the tumor microenvironment, where ferroptosis is pro-tumorigenic rather than anti-tumorigenic [20]. Transport of PUFAs, including AA, which are sensitive to lipid peroxidation, can be sufficient to induce ferroptosis in some contexts.

FATP family members transport selective fatty acids such that FATP1 and FATP2 exhibit strong selectivity for AA [32, 35]. Ferroptosis is induced by the peroxidation of PUFAs esterified in the phospholipids (especially PE) of membranes [36, 37]. In general, phospholipids containing PUFAs are known to increase sensitivity to ferroptosis because they contain many double bonds that are susceptible to peroxidation. Lysophosphatidylcholine acyltransferase 3 (LPCAT3), which shows selectivity for AA in fatty acyl remodeling of phospholipids, also promotes ferroptosis [38, 39]. T cell-derived interferon (IFN)-γ in combination with AA induces immunogenic tumor ferroptosis by activating AA to arachidonoyl-CoA by ACSL4 [40]. Altogether, the increase in phospholipids with AA, through the sequential action of FATP2 (uptake of exogenous AA), ACSL4 (activation of AA to its CoA form), and LPCAT3 (transfer of AA from arachidonoyl-CoA to lysophospholipids to give rise to AA-containing phospholipids), might greatly increase cellular sensitivity to ferroptosis.

We showed that the low expression of *SLC27A2* is possibly caused by the hypoxic signature of *BART* miRNA [41]. It has been reported that the winged helix/forkhead box (Fox) transcription factor FOXA1 regulates *SLC27A2* expression in the liver [42] and that FOXA1 is directly regulated by miR-4721, which is induced by EBV-miR-*BART22* [43, 44]. Thus, *SLC27A2* expression in Akata LCLs can be suppressed by *BART22*, which is deleted in B95-8 LCLs, through the miR-4721/FOXA1 axis. As a point to note, the protein expression values of FATP2 itself were similar between Akata LCLs and B95-8 LCLs (Supplementary Fig. 1E), indicating the existence of post-transcriptional regulation mechanisms of FATP2 expression. This unknown regulation might contribute to cellular resistance to ferroptosis. Further investigations were now ongoing.

In a subcutaneous xenograft model of tumor cells tested so far, the expression of *SLC27A2* regulated their viability and tumor size. Histological analysis revealed a prominent accumulation of lipid droplets just below the tumor capsule in the control tumor. This observation may be caused by the downregulation of the FATP family in Akata LCLs, which inhibits the uptake of external lipids into the tumor cells, resulting in the accumulation of lipids in the cells surrounding the tumor cells. In contrast, in *SLC27A2*-overexpressing Akata LCL tumors, there were few lipid droplets just below the capsule and massive infiltration of F4/80-positive cells was evident. Recent studies have shown that ferroptotic tumor cells can elicit an anti-tumor immune response [45], suggesting that ferroptotic Akata LCLs release certain damage-associated molecular patterns that induce a potent anti-tumor immune response. In line with this, chemotactic lipid mediators, such as 12-HETE [46], 20-HETE [47], and 12-HHTrE [48] were increased in *SLC27A2*-overexpressing tumor tissues.

Among tumors with *SLC27A2*^low^ expression, including high-grade tumors such as glioblastoma and melanoma, *SLC27A2*^low^ group had a significantly worse prognosis. Furthermore, in vitro and in vivo analyses using glioblastoma, melanoma, and HNSQ cell lines supported the anti-tumor effects of *SLC27A2*, suggesting that FATP2 can induce ferroptosis in several cancers, including EBV-infected lymphoma. The common or different mechanisms underlying these tumors should be further investigated.

Moreover, in silico analysis using the TCGA database revealed that *SLC27A2*^low^ tumors may have a poor prognosis. One possible interpretation of these results is that AA, a precursor of pro-inflammatory lipid mediators, may be decreased owing to the low expression of *SLC27A2* in tumor cells, followed by a failure to induce an appropriate anti-tumor immune response. Subcutaneous tumor tissues with wild-type Akata LCL showed few F4/80-positive inflammatory cells, suggesting an immunosuppressive phenotype that allows tumor development, whereas these inflammatory cells were markedly increased in tumor tissues with *SLC27A2*-overexpressed Akata LCL, resulting in tumor suppression.

Overall, we demonstrated that malignant tumors with low *SLC27A2*/FATP2 expression, such as EBV-positive lymphomas, acquire resistance to ferroptosis owing to a limited influx of exogenous AA, resulting in low intracellular AA levels despite high extracellular AA levels. These results highlight the potential of this novel pro-tumorigenic axis as a therapeutic target for cancer.

## Materials and methods

### Cell culture and preparation

Akata LCLs and B95-8 LCLs from an EBV-transformed umbilical cord blood B lymphoblast line, A-172 cells (RIKEN BRC, Tsukuba, Japan), THP-1 cells (ATCC Manassas, VA, USA), and HL-60 cells were cultured in RPMI 1640 medium (Wako, Osaka, Japan; #189-02025) supplemented with 10% (vol/vol) fetal bovine serum (FBS; ThermoFisher Scientific, MA, USA; #A5256701), 100 U/mL penicillin, and 100 mg/mL streptomycin (1% P/S) (Life Technologies, Carlsbad, CA; #15140122). PBMCs were isolated from whole blood samples of healthy 11 individuals using Lymphoprep™ (Abbott Diagnostics Technologies AS, Oslo, Norway; #07861). For B cells, the collected PBMCs were reacted with hCD19-Biotin antibody (clone HIB19; BioLegend, San Diego, CA, USA; #302203) for 20 min on ice and then reacted with Anti-Biotin MicroBeads (Miltenyi Biotec, Bergisch, Germany; #130-090-485) for another 20 min, which was separated and purified using autoMACS (Miltenyi Biotec). PBMC and B cells were used for assays immediately after collection. COLO679 cells (RIKEN BRC) and HSC-2 cells (RIKEN BRC) were cultured in RPMI supplemented with 20% FBS and 1% P/S, and High-glucose D-MEM (Wako; #043-30085) supplemented with 10% FBS and 1% P/S, respectively. Mycoplasma contamination was checked for all used cell lines by PCR.

### EBV

The Akata and B95-8 EBV strains were prepared as described previously [26]. For viral titration, 2 × 10^5^ cord blood lymphocytes per well in 6-well plates were inoculated with serial tenfold dilutions of the viral preparations. The 50% transforming doses were measured using the Reed–Muench method [26] after 6 weeks.

### Humanized mice

Following a previously reported method of humanization of NOG mice (Central Institute for Experimental Animals, Kanagawa, Japan) [49], 1 × 10^5^ CD34^+^ cord blood cells (RIKEN BRC) were administered intravenously after irradiation with 2 Gy X-rays. The ratio of human to mouse CD45^+^ cells in the peripheral blood indicates the efficiency of humanization. All the experiments were approved by the Institutional Review Board of Tokai University. The animals received human care as required by the institutional guidelines for animal care and treatment in the experimental investigations.

### Lipidomic analysis

MS-based lipidomics analysis was performed according to a previously published protocol [50]. For phospholipid detection, lipids were extracted from the exosomes and cells using the Bligh and Dyer method [51]. Electrospray ionization (ESI)-MS analysis was performed using 4000Q-TRAP, a triple quadrupole-linear ion trap hybrid mass spectrometer (Sciex, Framingham, MA, USA) with reverse-phase LC (NexeraX2 system, Shimadzu, Kyoto, Japan). The samples were injected by an autosampler, applied to a Kinetex C18 column (2.1 × 150 mm, 1.7 μm particle, Phenomenex, Torrance, CA, USA; #00F-4475-Y0) coupled to ESI-MS, and separated by a step gradient with mobile phase A (acetonitrile/methanol/water = 1:1:1 [v/v/v] containing 5 μM phosphoric acid and 1 mM ammonium formate) and mobile phase B (2-propanol containing 5 μM phosphoric acid and 1 mM ammonium formate) at a flow rate of 0.2 mL/min at 50 °C. To detect fatty acid metabolites, exosomes, and cells were soaked in methanol and water was added to a final methanol concentration of 10% (v/v). Samples in 10% methanol were acidified with ice-cold 1 N HCl solution to pH 3–4, applied to Oasis HLB cartridges (Waters, Milford, MA, USA; #41115712), washed with 5 mL hexane, eluted with 3 mL methyl formate, dried under N_2_ gas, and dissolved in 60% methanol. The samples were then subjected to ESI-MS, as described above. Sample separation was achieved using a step gradient with mobile phase C (water containing 0.1% acetic acid) and mobile phase D (acetonitrile/methanol = 4:1 [v/v]) at a flow rate of 0.2 mL/min at 45 °C. Lipids were identified using multiple reaction monitoring (MRM) transitions and retention times. Quantification was performed based on the peak area of the MRM transition and the calibration curve obtained using an authentic standard for each compound. As internal standards, d5-labeled EPA (#27358), LPC17:0 (#33331), and PE14:0-14:0 (#15090; Cayman Chemical, Ann Arbor, MI, USA) were added to each sample.

### Desaturase and elongase activities

Desaturase and elongase activities were calculated from the quantitative values of mass spectrometry as the ratio of products to precursors of individual fatty acids in the cell lines according to the following equation; $\Delta$-6 desaturase (D6D) = 18:3/18:2. $\Delta$-5 desaturase (D5D) = 20:4/20:3 [52, 53].

### Quantitative RT-PCR

Total RNA was isolated from the cultured cells using Sepasol-RNA I Super G (Nacalai Tesque, Kyoto, Japan; #09379-55). Quantitative RT-PCR (qPCR) was performed using a High-Capacity Reverse Transcription Kit (Applied Biosystems, Foster City, CA, USA; #4368814) and THUNDERBIRD SYBR qPCR Mix (TOYOBO, Osaka, Japan; QPX-201) following the manufacturer’s instructions. The expression of *FADS1, 2*, and *SLC27A* families were analyzed using qPCR, with *GAPDH* as an internal reference. The primers used are listed in Supplementary Data Table 1.

### Enrichment analysis of *BART*/THP-1 cells

*BART* clusters 1 and 2 were overexpressed in THP-1 cells using the Tet-off system (*BART*/THP-1). Ctrl/THP-1 and *BART*/THP-1 cells were cultured with 10% FBS for 4 days, and total RNA was harvested. Reverse-transcribed samples were hybridized to the whole human genome DNA microarray 4 × 44 K (Agilent Technologies, Santa Clara, CA, USA; #G4112F) and analyzed according to the manufacturer’s protocol. The obtained data were then analyzed using GSEA software [54, 55].

### Incubation under hypoxic conditions

THP-1 and HL-60 cells were incubated at 37 °C for 6 h under 1% O_2_ and immediately placed on ice and subjected to RNA extraction using Sepasol-RNA I Super G.

### Ferroptosis sensitivity test

For the ferroptosis sensitivity test, each cell was seeded in 96-well plates at 2 × 10^4^ cells/100 μL, and RSL-3 (Sigma-Aldrich, St. Louis, MO, USA; #SML2234) and Fer-1 (Cayman Chemical; #17729) were added to induce and inhibit ferroptosis, respectively. CB-2 (Sigma-Aldrich, St. Louis, MO, USA; #500670) was added 3 h before RSL-3 and AA was added simultaneously with RSL-3. After the addition of reagents, the cells were incubated at 37 °C and 5% CO_2_ for 16 h, followed by the addition of 10 Resazurin (Tokyo Chemical Industry, Tokyo, Japan; #R0195). Then, the cells were incubated for another 4 h under the same conditions, and the fluorescence was measured by GloMax® Multi+ (Promega, Madison, WI, USA; #E9032).

### Live cell imaging

Live cell staining with Liperfluo (Dojindo, Kumamoto, Japan; #L248) was performed according to the manufacturer’s protocols. Briefly, these dyes were diluted in PBS to 1 μM, added to the cell pellets, and incubated in the dark at 37 °C for 10–20 min. The supernatant was removed by centrifugation and the cells were washed three times with PBS. The stained cells were then resuspended in an appropriate volume of PBS and seeded on 4-well glass bottom dishes (Φ9.5 mm/well, MATSUNAMI, Bellingham, WA USA; #D141400). Using a confocal laser scanning microscope (LMS880, Carl Zeiss, Oberkochen, Germany), Liperfluo-stained cells were visualized by acquiring lipid peroxide-derived fluorescence spectra (520–550 nm) in the lambda mode and merging them with the transmitted light image to visualize their localization. Further, the positivity and cell diameter of Liperfluo-stained cells were quantified by BD FACSVerse™ Flow Cytometer (BD Biosciences, Franklin Lakes, NJ, USA). For HE staining, cell suspensions were applied to coated glass slides, allowed to stand for 30 min, immediately fixed in 99% ethanol for at least 5 min, washed with water, and stained with H&E. Images were visualized under a BX63 microscope (40× magnification; Olympus, Tokyo, Japan) and calculated using ImageJ software.

### Lentiviral transfer

*SLC27A2* cDNA amplified from the 293T cell line was cloned into the multiple cloning site of the CSII-CMV-MCS-IRES2-Venus vector using the restriction enzymes NotI (New England Biolabs, Ipswich, MA, USA; #R0189) and BamHI (New England Biolabs, Ipswich, MA, USA; #R0136). High-titer lentiviral supernatants were observed after cotransfection of *SLC27A2*-vector and pCAG-KGP1R for gag protein, pCAG-4RTR2 for Rev/tat, and pCMV-VSVG viral packaging constructs into HEK293T cells using PEI MAX-Transfection Grade Linear Polyethylenimine Hydrochloride (Polysciences, Inc. Warrington, PA, USA; #24765-100). The cells were plated at a concentration of 4–5 × 10^5^/mL in a 24-well plate and spin-infected with 500 μl of lentiviral supernatant (32 °C, 2500 rpm for 99 min). Plates were incubated overnight at 37 °C in humid air containing 5% CO_2_. Three days after transduction, the transduced cells identified as GFP^+^ or Venus^+^ were purified using a FACS Aria (BD Biosciences).

### Western blotting

Proteins were separated using sodium dodecyl sulfate-polyacrylamide gel electrophoresis and transferred onto polyvinylidene fluoride membranes. The membranes were then incubated with mouse monoclonal anti-human FATP2 antibody (Abcam, Cambridge, UK; #ab175373; 1:500 dilution) and rabbit polyclonal anti-GAPDH antibodies (Sigma-Aldrich, #G9545; 1:1000 dilution). Horseradish peroxidase-conjugated goat anti-mouse and anti-rabbit IgG polyclonal antibodies (Proteintech, Rosemont, IL, USA; #SA00001-1 and #SA00001-2, respectively). Protein signals were detected using Ez-Capture MG AE-9300 (ATTO, Tokyo, Japan).

### Development of NOG mouse xenograft model

NOG mice (6 weeks old, 16–18 g) were purchased from the Central Institute for Experimental Animals (Kanagawa, Japan). *SLC27A2*-overexpressing Akata LCLs and empty vector Akata LCLs were injected subcutaneously into the left and right shoulder at 1 × 10^6^ cells/50 μl. After injection, the animals were maintained on a normal diet, and the tumors were surgically removed from under the skin after 5 weeks.

A172 cells (2 × 10^5^ cells/5 μl) were intracranially injected into the right frontal lobe at a depth of 1 mm and the brains were removed 3 weeks later. The animals received humane care according to the institutional guidelines for animal care and treatment in experimental investigations.

### Analysis of subcutaneous tumor tissue

The harvested subcutaneous tumors were weighed and halved in the longitudinal direction; one was fixed in 10% formalin and made into a permanent specimen, while the other was quickly frozen in liquid nitrogen with an optimal cutting temperature compound to make a frozen block. The frozen blocks were sectioned in a cryostat, and some sections were collected, washed with PBS, and analyzed by mass spectrometry after lipid extraction. Other sections were used for histological analyses, such as immunostaining.

### Histological analysis of tumor tissue

Permanent specimens of subcutaneous tumor tissues were H&E stained and visualized using a BX63 microscope (Olympus) or NanoZoomer S360 (Hamamatsu Photonics, Tokyo, Japan). Immunohistochemistry using anti-Ki-67 (MIB-1) (clone MM1; Leica, Wetzlar, Germany; #ACK02), rabbit polyclonal anti-Melan A (Proteintech, IL, USA; #18472-1-AP), and anti-FATP2 antibodies, and in situ hybridization using a probe against EBER (Bond ISH EBER; Probe, #PB0589) were performed using a Bond Max autostainer (Leica, Wetzlar, Germany). The MIB-1 index and positivity rates for Melan A were determined in whole tissue areas, visualized under a BX63 microscope and NanoZoomer S360, and calculated using ImageJ software. Fluorescent double immunostaining using FITC-labeled anti-mouse CD19 antibody (clone eBio1D3(1D3); Invitrogen; #11-0193-82), APC-labeled anti-mouse F4/80 antibody (clone BM8; BioLegend; #123115), and lipid peroxide staining with Liperfluo were performed on the frozen sections. Before staining, specimens for immunostaining were fixed with cold methanol, and specimens for Liperfluo staining were fixed by dry fixation. Each observation was performed using an LSM880 confocal laser microscope (Leica) and the obtained images were analyzed with ImageJ.

### Database analysis

Using UCSC XENA (https://xenabrowser.net/datapages/), a cancer genomics data analysis platform that includes data from TCGA and other sources, as well as a tool that can provide online analysis and data visualization [52], we extracted the gene expression data of *SLC27A2* and disease-specific survival data of all cancer patients from TCGA and analyzed the prognosis of the high *SLC27A2* expression group and the low *SLC27A2* expression group. Immune Model Based subtype data and the presence or absence of *SLC27A2* mutations in the above dataset were also extracted on the same tool to visualize the relationship with *SLC27A2* expression and prognosis, respectively. This dataset was also projected onto a Tumor Map (https://tumormap.ucsc.edu/) to visualize the tumor histological types of the *SLC27A2* high-expressing and low-expressing groups, respectively. In addition, the normal tissues and tumor tissues at the same primary site were examined in the same manner by use of gene expression data for normal tissue samples extracted from the GTEx study.

### Statistical analyses

Statistical significance was obtained using the two-sided Student’s *t*-test. The variance similarities between the compared groups were confirmed using the *F*-test. Statistical significance was set at *p* < 0.05 was statistically significant. Survival curves were analyzed using the log-rank test and EZR software (http://www.jichi.ac.jp/saitama-sct/SaitamaHP.files/statmed.html) [53]. All experiments were independently replicated more than two times. Sample sizes for all experiments were determined empirically from previous experimental experience with similar assays. Specific sample sizes and the count of independent experiments conducted for each study can be found in the figures, their accompanying legends, or within the “Materials and Methods” section. All samples were included in the analyses.

### Study approval

All experiments were approved by the Institutional Review Board of Tokai University. Written informed consent was obtained from all patients and healthy volunteers.

### Supplementary information

Noncropped Western Blotting membrane of Supplemental Figure S1E

Supplementary Information

Supplementary Data Table 1

## Data Availability

The data supporting the findings of this study are available in the article and its Supplemental materials. Raw data are provided as Source Data files or are available from the corresponding author upon request.
