# Supplementary figures and images for: Unique lipid composition maintained by extracellular blockade leads to prooncogenicity
Source: Cell Death Discov. 2024 May 8;10:221. doi: 10.1038/s41420-024-01971-y (PMC11079073; doi:10.1038/s41420-024-01971-y)

Non-edited membrane of western-blot in Fig. S1E

GAPDH

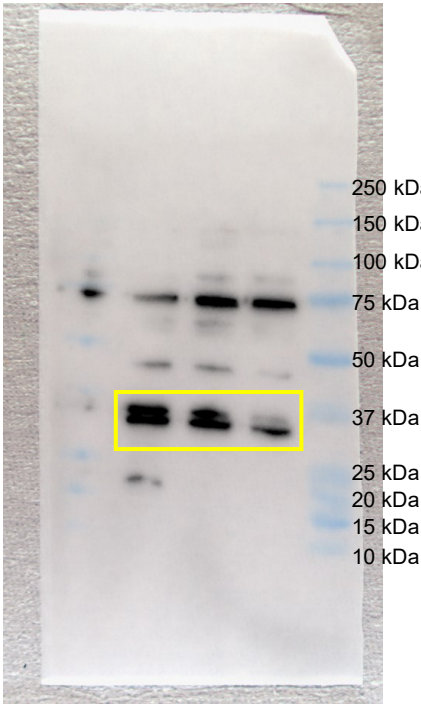

FATP2

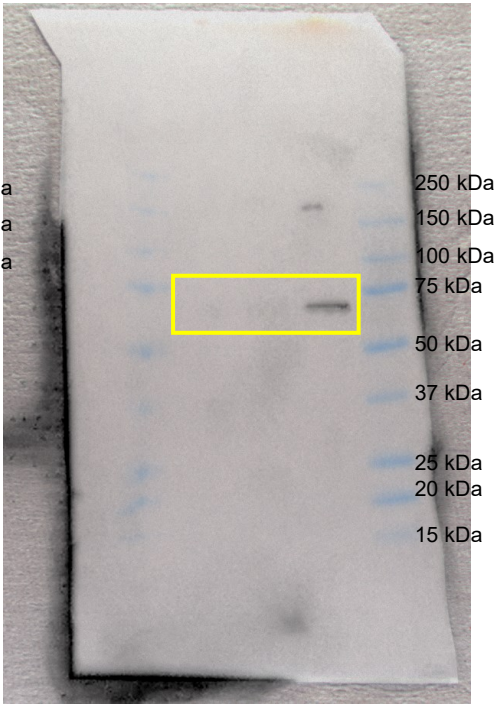

FATP2 long exposure

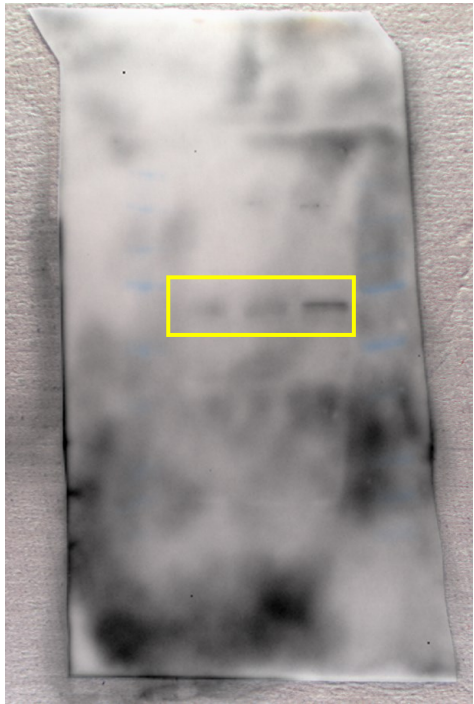

Supplement: Supplementary file 1 — Noncropped Western Blotting membrane of Supplemental Figure S1E [file 41420_2024_1971_MOESM1_ESM.pdf]
