# Supplementary material for: Unique lipid composition maintained by extracellular blockade leads to prooncogenicity
Source: Cell Death Discov. 2024 May 8;10:221. doi: 10.1038/s41420-024-01971-y (PMC11079073; doi:10.1038/s41420-024-01971-y)
Supplement: Supplementary file 2 — Supplementary Information [file 41420_2024_1971_MOESM2_ESM.pdf]

SUPPLEMENTAL FIGURE

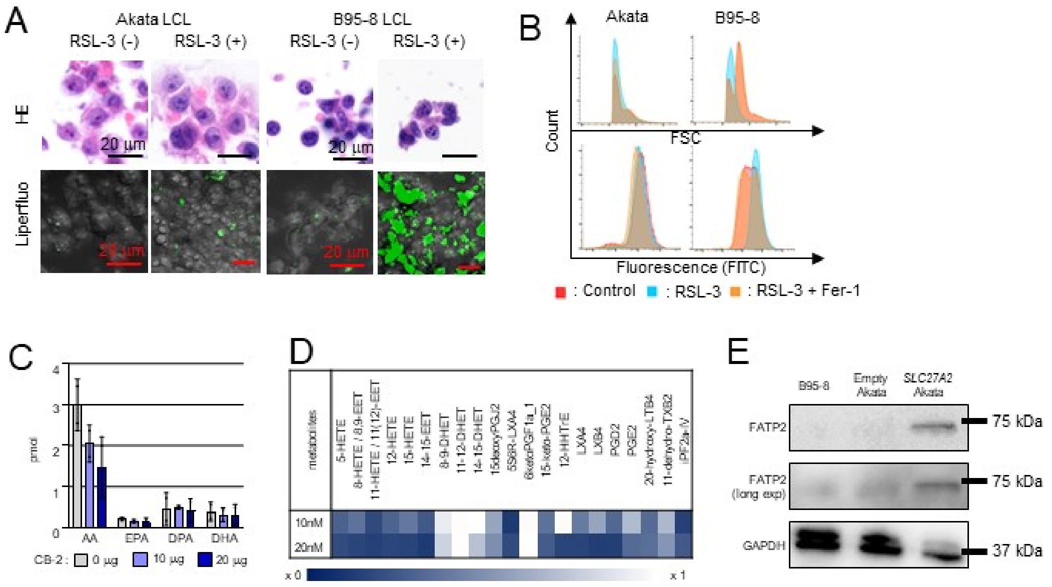

Supplemental Fig. S1. AA influx via FATP2 increase ferroptosis sensitivity in LCL.

A. Morphological changes (upper) and lipid peroxidation status (lower) of LCLs exposed to RSL-3.

B. Measurement of fluorescent intensities of Liperfluo in supplemental figure 1A by flow cytometer.

C. Intracellular lipid concentrations of B95-8 exposed to CB-2.

D. Relative intracellular composition of AA metabolites of B95-8 exposed to CB-2, compared with those of CB-2 non-exposed B95-8 cells.

E. Western Blotting analysis of FATP2 (upper) and GAPDH (lower) in B95-8, *SLC27A2*-overexpressed Akata, and native Akata.

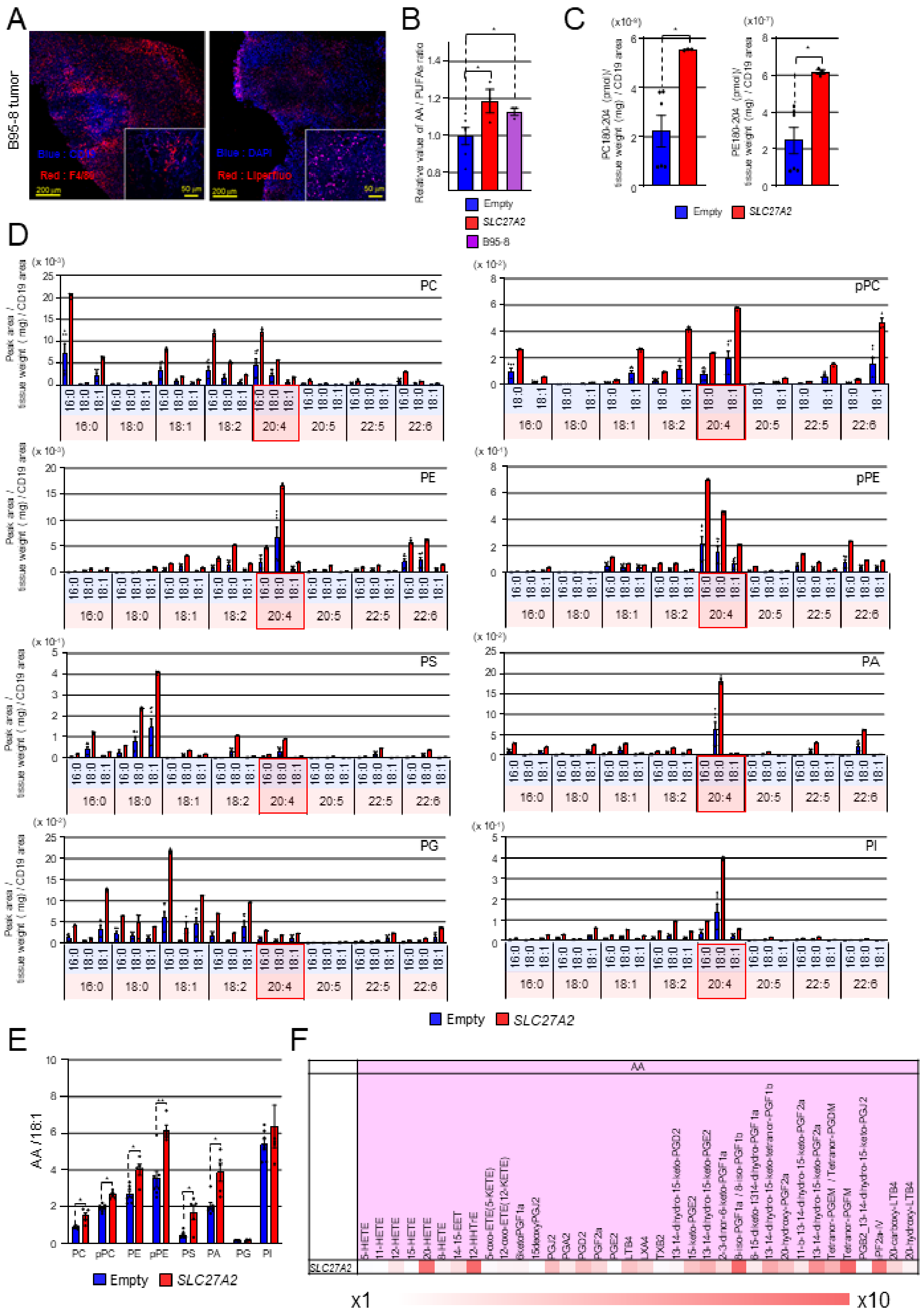

Supplementary Fig. S2. Analysis of xenograft tumor model tissue of B95-8 LCL and SLC27A2 overexpressed Akata LCL.

A. Immunostaining and Liperfluo staining in B95-8 LCL tumor tissues. Frozen sections of tumor tissues were stained with hCD19, mF4/80, Liperfluo, and DAPI. In immunostaining, blue signal indicates hCD19-positive cells and red signal indicates

mF4/80-positive cells; in Liperfluo staining, red signal indicates the presence of lipid peroxide and blue signal indicates nuclei (DAPI).

B, C. Quantification of major AA in tumor tissue. Lipid extraction was performed from tumor tissue collected from frozen sections. Each quantitative value was calculated from the calibration curve and shown as the relative amount of (**B**) free AA, and (**C**) stearoyl-arachidonoyl PC and PE to the amount of tumor cells ($n = 3$).

D. ESI-MS analysis of the phospholipid molecular species in xenograft tumor tissues. Each peak area values are compensated with tumor cell amount ($n = 3$).

E. AA/18:1 ratio in each phospholipid headgroup in mass spectrometry. The ratios were calculated from the peak area values of AA-bound phospholipids and 18:1-bound phospholipids in each headgroup ($n = 6$).

F. Amount of AA metabolites in tumor tissues. Lipids were extracted from frozen sections and peak area values were compensated with tissue weight. Heat maps are shown as relative values to Empty tumors ($n = 3$).

Values in (B, C, D, and E) represent the mean ± SEM. Student's t-test: *$p < 0.05$, **$p < 0.01$, and ****$p < 0.0001$

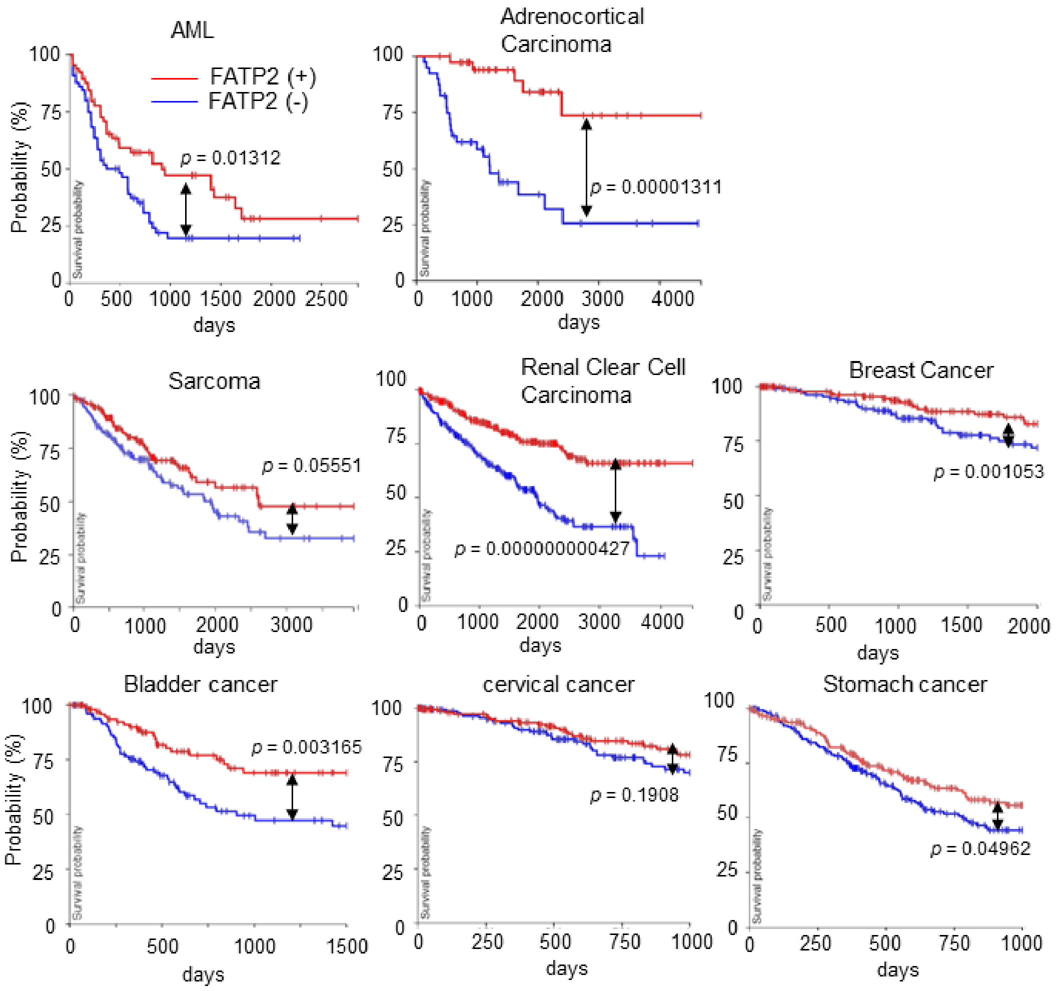

Supplementary Fig. S3. Analysis of FATP2 expression and prognosis in various malignancies.

Relationship between FATP2 expression and prognosis in other histopathologic type of malignant tumors extracted from the TCGA database. The red and blue lines indicate cases in the FATP2 high and low expression groups, respectively. The number of cases is shown as 132 for AML, 79 for adrenocortical carcinoma, 265 for sarcoma, 602 for renal clear cell carcinoma, 1,214 for breast cancer, 426 for bladder urothelial carcinoma, 299 for cervical cancer, and 443 for stomach cancer, respectively. The analysis was performed on UCSC Xena.

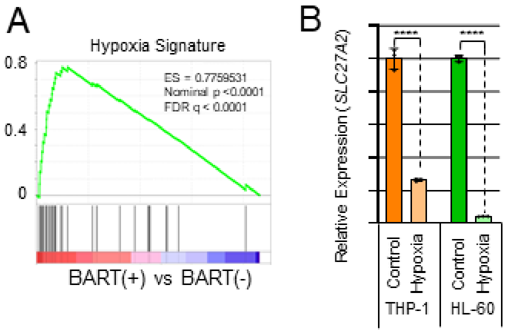

Supplemental Fig. S4. BART miRNA-overexpressed THP1 shows hypoxic phenotype, in which *SLC27A2* expression is up-regulated.

A. Gene set enrichment analysis about hypoxia signature of BART miRNA-overexpressed THP1 compared with native THP1 cells, by gene set enrichment analysis.

B. Quantitative PCR of *SLC27A2* in THP-1 and HL-60 cultured under normoxic (Control) and hypoxic condition.

Values in (B) are presented as mean ±SEM. Student's t-test: ****p<0.0001.

Non-edited membrane of western-blot in Fig. S1E

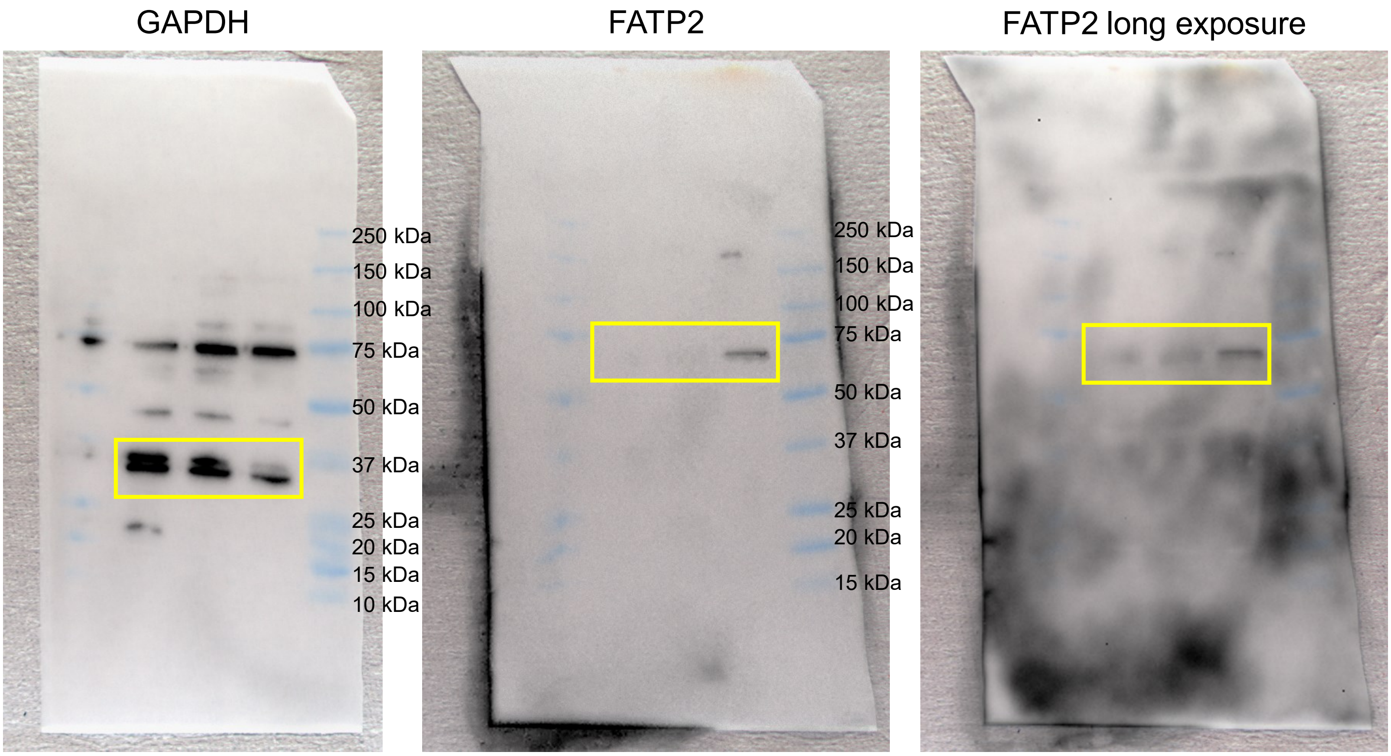

Source data of Figure 1A

Target	NC mouse serum	NC mouse serum2	NC mouse serum3	EBV mouse serum	EBV mouse serum2	EBV mouse serum3	FC	Normalized_NC mouse serum	Normalized_NC mouse serum2	Normalized_NC mouse serum3	Normalized_EBV mouse serum	Normalized_EBV mouse serum2	Normalized_EBV mouse serum3
14 1 CE5	1.931776536	3.106039573	3.457900298	10.69378101	6.392170399	12.10254956	3.435673	0.307573303	0.494536937	0.550559444	1.702640801	1.017747616	1.926941899
17 0 CE5	11.81440685	13.49348203	13.49663595	39.05965634	30.96673456	40.52578013	2.848951	0.474611739	0.542064028	0.542190728	1.569115711	1.244004538	1.628013256
AA	3.068186802	4.177425036	4.14681539	10.91448734	9.449226047	11.39459043	2.787668	0.426623614	0.580860384	0.576604191	1.517631856	1.31389098	1.584388976
13 0 CE5	1.178284371	1.396285496	1.448871823	3.986195299	3.165066454	3.922321018	2.752266	0.468284744	0.55492478	0.575824127	1.584230843	1.257890174	1.558845332
20 2 CE5	14.72416855	15.37878441	14.25029794	39.60591571	31.44489114	41.84726717	2.54543	0.561807739	0.586784922	0.543726978	1.511182779	1.199794958	1.596702623
20 3 CE5	19.73063859	22.14763138	21.00076143	55.94988954	45.50652459	56.87455963	2.518025	0.535164906	0.600722323	0.569615142	1.517559466	1.234298364	1.542639798
15 1 CE5	0.670472104	1.054845865	0.965912047	1.967166387	1.544070835	1.829718554	1.984578	0.50083909	0.787964242	0.721531154	1.469462812	1.153412688	1.366790013
DPA n-6	1.05234841	1.213663777	1.187900709	2.333593893	1.996533443	2.502591866	1.978255	0.613815134	0.707907369	0.692880253	1.361141647	1.164540594	1.459715002
18 0 CE5	201.4541527	225.9859024	213.7806461	459.3417753	355.4283744	452.1843495	1.975848	0.633445459	0.710582243	0.672204458	1.444338367	1.117596668	1.421832805
18 1 CE5	353.8351915	460.4389402	433.9205539	832.314244	692.943583	875.1338061	1.923091	0.581872255	0.757179192	0.713570434	1.368717916	1.139526692	1.43913351
15 0 CE5	6.232447535	7.643384644	7.132615921	13.7637664	11.18486607	13.08668494	1.810477	0.633338421	0.776717194	0.724813114	1.398667543	1.13660089	1.329862839
DHA	49.14150548	55.32196195	53.05744892	101.3763329	80.74026716	102.8956989	1.809362	0.666275485	0.750071995	0.719369038	1.374491171	1.094701112	1.395091198
rLN 1	0.194815471	0.201144485	0.181108679	0.454061607	0.257806149	0.309564727	1.770036	0.731243046	0.754999103	0.67979438	1.704327641	0.96767958	1.16195625
12 0 CE5	4.091502079	4.797018619	5.399662251	9.228015858	7.151662826	8.439369243	1.737033	0.627735892	0.735979282	0.828439467	1.415801986	1.09723895	1.294804422
rLN 2	0.064248353	0.107587749	0.109559639	0.184868521	0.14307564	0.159106753	1.73084	0.50164851	0.840040736	0.855437172	1.44344584	1.117128735	1.242299007
pLPC140	0.000325686	0.000318802	0.000441476	0.000465148	0.000866779	0.000498789	1.685798	0.669980146	0.65581889	0.908174225	0.956871971	1.783079932	1.026074836
14 0 CE5	27.07317195	33.03745764	30.96729014	54.20266298	44.14880077	52.78086545	1.659374	0.670653007	0.818399497	0.767117583	1.342701142	1.093648208	1.307480563
16 1 CE5	87.93840102	105.0457019	99.15551556	171.6453355	137.0149944	170.7045813	1.640876	0.683898002	0.816941686	0.771133636	1.334887838	1.065567258	1.32757158
aLN rLN	0.431083905	0.482981342	0.433766058	0.835150542	0.595250844	0.750565342	1.61813	0.732970095	0.821211083	0.737530549	1.420002847	1.012102429	1.276182998
16 0 CE5	294.5504339	331.8685748	317.4294746	517.5495825	432.1943206	538.1198528	1.57638	0.72677292	0.818851596	0.783224601	1.277000397	1.066395062	1.327755425
18 3 CE5	38.93621792	45.53827315	43.30395119	70.16569124	55.43115676	69.58640035	1.527513	0.723359192	0.846012536	0.80450318	1.30354206	1.02980307	1.292779962
pLPC200	0.035234284	0.032571255	0.032613708	0.048099438	0.052848404	0.049974576	1.502923	0.84110888	0.77753735	0.778550773	1.148224377	1.261591161	1.192987459
LPI204	0.012671933	0.009245513	0.012747364	0.01581525	0.017335796	0.018811604	1.499003	0.877684705	0.640363651	0.882909199	1.095397476	1.200713706	1.302931263
DPA n-3	2.310863593	3.226804267	2.753665985	4.360567352	3.535279234	4.424516325	1.485933	0.672685113	0.939312558	0.801583494	1.269347421	1.029108649	1.287962765
18 2 CE5	391.0338735	388.2573357	369.7802035	577.1983743	472.0049084	605.5334746	1.440064	0.836791642	0.830849999	0.791309921	1.235173748	1.010065339	1.295809352
20 1 CE5	98.7299169	99.26128615	94.96713621	154.4089023	117.7244866	148.2142211	1.434837	0.830470434	0.834940067	0.798819662	1.298816328	0.990243977	1.246709532
LPA202	0.001125063	0.000690729	0.001324431	0.001302422	0.001251997	0.001612242	1.326867	0.923838468	0.567187974	1.087547708	1.069475257	1.028068897	1.323881695
pLPI170	0.002457503	0.001248551	0.001986684	0.002604869	0.001878105	0.002399092	1.20892	1.172584402	0.595739533	0.947935596	1.242899039	0.896127765	1.144713665
LPS205	5.92136E-05	5.3133E-05	0.000165561	9.30367E-05	9.631E-05	0.000142521	1.194166	0.5826431	0.522812029	1.62906931	0.915451568	0.947659333	1.40236466
pLPA181	0.001125081	0.001992519	0.001710707	0.002069964	0.001565001	0.002066448	1.180831	0.641089077	1.135368696	0.974787488	1.179498123	0.891762332	1.177494285
pLPG180	0.000828964	0.000903266	0.000441463	0.000790752	0.001155734	0.000617546	1.179574	1.049825177	1.14392349	0.559082255	1.00143257	1.463657786	0.782078722
20 5 CE5	36.93847678	40.12528512	38.84002706	48.79602587	37.52334646	49.31605143	1.170241	0.88109865	0.957114035	0.926456594	1.163938426	0.895049628	1.176342668
pLPC181	0.005743979	0.005951001	0.00562874	0.006070416	0.007174813	0.006721834	1.152585	0.924192836	0.957502197	0.9056512	0.97671573	1.154410587	1.081527451
LPC202	0.161945076	0.14377956	0.142289167	0.167400162	0.168257116	0.169207696	1.126896	1.019720955	0.905337993	0.895953425	1.054070041	1.059466031	1.065451556
LPG170	0.000592141	0.000478209	0.000165557	0.0002791	0.000409315	0.000665074	1.095138	1.372074752	1.108079639	0.383619234	0.646713488	0.948440528	1.541072359
EPA	6.840656807	6.661432438	6.911192351	7.818311314	6.230510606	8.290807541	1.094367	0.96002681	0.934874226	0.969925862	1.097232135	0.874398087	1.16354288
LPC204	0.989873061	0.842887765	0.879683865	0.939188061	0.982183653	1.030648255	1.088324	1.048508327	0.892816337	0.93179206	0.994820994	1.040363436	1.091698846
pLPI151	0.00065135	0.000292238	0.00038631	0.000697729	0.000264844	0.00047505	1.081003	1.412130375	0.633573197	0.837521277	1.512681276	0.574183739	1.029910136
LPG161	0.006839275	0.007226105	0.006842721	0.009140216	0.007319419	0.005985441	1.073511	0.946543122	1.000079603	0.947020035	1.264989093	1.012994097	0.828374049
LPC181	4.762724429	4.630686477	4.692054587	5.027160586	5.067162866	4.701393203	1.050424	0.989445184	0.962014599	0.974763684	1.044381195	1.052691577	0.976703761
pLPI183	0.001421172	0.001062677	0.00060702	0.001162927	0.000746376	0.001330127	1.048064	1.347019032	1.007229119	0.575347159	1.102248566	0.707431728	1.260724396
LPA200	0.000422936	0.00092983	0.000441463	0.000604703	0.001131645	0.000142521	1.047174	0.69086564	1.518875695	0.721128794	0.987781564	1.848539919	0.232808388
pLPI201	0.001421168	0.000956407	0.000220748	0.000604693	0.00134831	0.000760074	1.044165	1.605415954	1.080400809	0.249367357	0.683088399	1.52311298	0.8586145
pLPC150	0.016936204	0.015727711	0.01611428	0.016467334	0.016468412	0.0175771	1.035562	1.02342791	0.950400595	0.973760375	0.995094858	0.995159985	1.062156277
pLPA120	0.000118436	0.000318802	0.000441442	0.000465152	0.00014447	0.000285028	1.018175	0.400724833	1.078655773	1.493602382	1.573823755	0.488810576	0.964382681
alkylLPA18	0.00373049	0.004569457	0.005463137	0.004163014	0.003322759	0.006318009	1.002957	0.811950847	0.994554221	1.189065989	0.906090785	0.723207033	1.375131124
LPI205	0.001065868	0.00053133	0.001489937	0.00102334	0.001155716	0.000902567	0.998214	1.036708981	0.51679478	1.449177208	0.99534469	1.124099246	0.877875095
LPC180	5.391044912	5.472601386	5.408181429	5.42689264	5.419706122	5.393431831	0.998046	0.994906817	1.009957905	0.998069328	1.001522445	1.000196187	0.995347318
LPS202	0.000473709	0.000371939	0.000331114	0.000418644	0.000601938	0.000142521	0.988393	1.21470803	0.95374524	0.849058912	1.073507573	1.543519894	0.365460351
pLPE140	0.000204989	0.00014649	1.63093E-05	3.33332E-05	0.000143525	0.000185874	0.986252	1.683640783	1.203170231	0.133953049	0.273775392	1.178817378	1.526643168
pLPI141	0.008527291	0.009085834	0.005491055	0.007303081	0.008354743	0.007102174	0.985103	1.115549126	1.188618368	0.718345566	0.955396805	1.092976264	0.929113871
LPI226	0.00319768	0.003719381	0.003145432	0.003209685	0.00346706	0.003230332	0.984555	0.960765845	1.117514624	0.945067354	0.964372875	1.041702922	0.970576379
LPA181	0.027416365	0.024920155	0.024113964	0.022141379	0.02756863	0.023703476	0.960275	1.097650031	0.997711003	0.965434102	0.88645905	1.103746154	0.948999659
LPC141	0.015811118	0.015302622	0.016665944	0.014792673	0.013771806	0.017315865	0.960248	1.012883592	0.980308611	1.067645072	0.947640512	0.882242251	1.109279962
LPG181	0.006928134	0.006853894	0.006097978	0.006093498	0.006861815	0.005605663	0.93365	1.081366894	1.069779219	0.951793249	0.951093949	1.071015591	0.874951098
LPI180	0.028719006	0.029435847	0.030076538	0.030955242	0.026846623	0.024466032	0.932411	1.01064373	1.035869911	1.058416307	1.089338597	0.944753131	0.860978325
LPS161	0.000888227	0.000584484	0.000331097	0.000744244	0.00057786	0.000356278	0.930466	1.530462723	1.007096672	0.570498205	1.282372585	0.995683573	0.613886242
pLPG141	0.008882401	0.009723398	0.009878078	0.008838021	0.01042524	0.007125714	0.926453	0.971234462	1.063192211	1.080105459	0.966381791	1.139934247	0.77915183
LPS200	0.001065918	0.000956403	0.001489992	0.000860583	0.001300154	0.001092606	0.926268	0.945289969	0.848168717	1.321372884	0.763192367	1.153017976	0.968958087
LPS160	0.001480449	0.001487737	0.00256614	0.001721155	0.001444686	0.001947767	0.92398	0.834217635	0.838324394	1.445993163	0.969852716	0.814065773	1.097546319
LPI203	0.006484188	0.004994351	0.005711828	0.005395953	0.005682421	0.004655673	0.915283	1.181649794	0.910148541	1.040898398	0.983334627	1.035539343	0.848429297
LPA182	0.091963603	0.078319519	0.084489287	0.076800285	0.078734843	0.075961886	0.908642	1.134724065	0.966371921	1.042499683	0.947626334	0.97149653	0.937281466
LPC203	0.60943395	0.53133276	0.550800287	0.528554213	0.513247422	0.494983116	0.908498	1.132653436	0.987499755	1.023680807	0.982335731	0.953887548	0.919942722
LPG180	0.000355309	0.000159403	0.000441463	0.000325629	0.000385225	0.000142521	0.892489	1.178112264	0.528539638	1.463776824	1.079700028	1.277307225	0.472564022
LPE170 a	0.0016579	0.000903274	0.001545129	0.000976772	0.001733624	0.000902567	0.879858	1.288646199	0.702093077	1.200991501	0.759221591	1.347504246	0.701543386
LPS201	0.001006672	0.000717314	0.000882938	0.000604707	0.000625991	0.00099761	0.854765	1.249171313	0.890109258	1.095631007	0.750375655	0.776787048	1.237925719
LPG182	0.003849026	0.004835426	0.00446974	0.00453542	0.00320219	0.003491368	0.853643	0.947135007	1.189859948	1.099875119	1.116036939	0.787967319	0.859125669
LPS204	0.006513625	0.006057275	0.006429088	0.004605041	0.005176683	0.006223107	0.84236	1.116467746	1.038247095	1.101977578	0.78932695	0.88730911	1.066671521
pLPC180	0.142172688	0.127466717	0.130729071	0.110403441	0.115332367	0.110636595	0.840157	1.157850953	1.038085878	1.064654406	0.899122971	0.939264023	0.901021769
pLPC183	0.000710582	0.000159407	0.000386263	0.000139555	9.63137E-05	0.000807581	0.830606	1.853931265	0.415898776	1.00777372	0.364103929	0.251285563	2.107006747
pLPE161	0.000651363	0.000159403	0.000441467	0.000465159	0.000288934	0.000285021	0.829808	1.705625724	0.417404645	1.156002364	1.218040143	0.756586224	0.7463409
LPE204	0.044472211	0.040224216	0.039373068	0.032330011	0.035633359	0.034155069	0.823075	1.179697169	1.067012245	1.044434174	0.857605713	0.945232372	0.906018329
pLPI182	0.001658004	0.000797024	0.000910536	0.001162874	0.000481506	0.001116349	0.820287	1.623824617	0.780593365	0.891765628	1.138901473	0.47157966	1.093335257
pLPI181 a	0.000651372	0.000265677	0.000331097	0.000139552	0.000674148	0.000190018	0.804166	1.735555454	0.707886444	0.882194976	0.371829409	1.796239463	0.506294254
LPI181	0.007875448	0.00696065	0.005352759	0.00618647	0.004767236	0.005272976	0.803744	1.29759688	1.146870351	0.88194637	1.019312726	0.785472752	0.868800922
pLPI160 a	0.004204271	0.002948971	0.003448935	0.00286078	0.003274455	0.002375171	0.802704	1.319843963	0.925768266	1.082721847	0.89808267	1.027947325	0.745635928
LPC182	12.09258502	11.14037987	11.45791312	9.376823896	9.267148077	9.089149864	0.799436	1.162301524	1.070778537	1.101298842	0.901271042	0.890729344	0.873620712
pLPG170	0.018948821	0.018490365	0.018542339	0.013652384	0.013290002	0.01676962	0.780829	1.140424305	1.11283238	1.11596039	0.821661172	0.799851433	1.00927032
pLPI161	0.001806068	0.002417571	0.002759215	0.001418775	0.001829868	0.002185248	0.778176	0.872725197	1.168215119	1.333303531	0.685578098	0.884225822	1.055952233
LPC160	16.99114693	15.36220301	15.74363091	12.29891627	12.63540845	12.35140006	0.77522	1.193999193	1.079530303	1.106333944	0.864267501	0.887913426	0.867955634
LPI182	0.008763874	0.006482323	0.005187416	0.005581856	0.00601939	0.004228004	0.774667	1.450057687	1.072555678	0.858302279	0.923565651	0.995959442	0.699559264
LPS180	0.007994192	0.007970065	0.005435859	0.005023749	0.005296986	0.006009593	0.763095	1.271258561	1.267421789	0.864425428	0.798890519	0.842341486	0.955662217
LPE202	0.001657887	0.002125362	0.001269206	0.00144191	0.001252048	0.001140084	0.758847	1.119374821	1.435005555	0.856944624	0.973551043	0.845360019	0.769763937
pLPI150	0.064899421	0.060306095	0.060897078	0.044189688	0.048104607	0.048265906	0.755283	1.192044307	1.1076761	1.118531014	0.811656954	0.883564481	0.886527144
LPE151 p	0.041980469	0.038469217	0.036393143	0.030000786	0.027738217	0.029048408	0.742771	1.236961738	1.13350208	1.072330203	0.883978312	0.817311336	0.855916331
LPA226	0.003582594	0.004197522	0.004856273	0.00311661	0.003659559	0.002588993	0.741126	0.977002159	1.144698016	1.324344742	0.849924564	0.997991171	0.706039348
LPC150 a	0.28678551	0.266316544	0.28066272	0.199302474	0.203015311	0.214836522	0.740202	1.18594695	1.101301432	1.160627319	0.824177489	0.839531222	0.888415589
20 0 CE5	16.61737635	14.76748267	14.4075076	12.11138967	9.583092842	12.07876833	0.73753	1.253107326	1.113607848	1.086462323	0.913313318	0.722655827	0.91085336
pLPE200	0.006691244	0.003506942	0.005573743	0.004511983	0.004141193	0.002897862	0.73238	1.469366816	0.770108676	1.223968693	0.990811097	0.909387296	0.636357422
LPC225	0.227386981	0.213438869	0.212957396	0.159806766	0.159774984	0.156529203	0.72824	1.207477556	1.133409848	1.130853117	0.848610957	0.848442187	0.831206336
pLPA180	0.001480345	0.001354903	0.001407182	0.00083727	0.000722338	0.001472645	0.714744	1.220956538	1.117494161	1.160613114	0.690562296	0.595768448	1.214605443
LPE180	0.06253233	0.052312303	0.054192211	0.039679398	0.036355686	0.044608173	0.71371	1.295201092	1.083518745	1.122456339	0.821859646	0.753017262	0.923946916
LPA180	0.027623579	0.031720863	0.030047336	0.020420455	0.020754208	0.02166189	0.702934	1.088768901	1.250261227	1.18430004	0.804861529	0.818016227	0.853792076
LPG140	0.002250195	0.001009556	0.001765906	0.001069873	0.001540912	0.000855078	0.689634	1.589959023	0.713339519	1.247767093	0.75595851	1.088788885	0.604186971
LPS225	0.002427808	0.002072282	0.0021797	0.000930342	0.001444606	0.001947629	0.647113	1.323974201	1.130092217	1.188671468	0.507350334	0.787797184	1.062114595

pLPE201	0.001687613	0.000478197	0.001434805	0.000883781	0.000866786	0.000570053	0.644507	1.710061735	0.48455827	1.453890668	0.895537516	0.878316097	0.577635714
LPC201	0.372574239	0.355992932	0.361672199	0.245250682	0.228661745	0.223743532	0.639911	1.250322319	1.194677091	1.213736151	0.823037048	0.767366214	0.750861176
LPE161	0.001598763	0.001009548	0.00096577	0.000604675	0.000818655	0.000855121	0.637493	1.639047971	1.034985841	0.990104382	0.619911061	0.839282959	0.876667786
LPI201	0.000769818	0.000956431	0.001158912	0.000372133	0.00091491	0.00052256	0.62721	0.983842264	1.222337742	1.481111917	0.475592926	1.169272862	0.66784229
LPC161 p	1.163477169	1.07781282	1.086395029	0.683013606	0.699527473	0.696537657	0.624782	1.291135202	1.196071664	1.205595523	0.757954632	0.77628042	0.772962558
pLPE181	0.013708463	0.014687574	0.017658609	0.008698345	0.010497736	0.009073194	0.61382	1.1066528	1.185694204	1.425539078	0.702197481	0.84745823	0.732458206
LPS181	0.004677967	0.004675849	0.002704007	0.001604749	0.00298565	0.002707732	0.605261	1.450086218	1.449429706	0.838193793	0.497443464	0.925498149	0.83934867
LPE181	0.019541066	0.026035912	0.023977003	0.012861877	0.01531253	0.013871059	0.604501	1.050600137	1.399787175	1.289092554	0.691502186	0.823258396	0.745759552
pLPE180	0.019511168	0.014345984	0.020142217	0.011349693	0.011460862	0.009477197	0.597928	1.356714711	0.997552183	1.40059489	0.789204207	0.796934367	0.658999643
LPG160	0.004618694	0.005697442	0.004928442	0.004674923	0.003322641	0.001068849	0.59473	1.139902786	1.406139835	1.216349153	1.153779993	0.820034285	0.263793948
LPC140	0.155444462	0.141758097	0.162293812	0.092894209	0.094690196	0.08377254	0.590553	1.276134009	1.163774678	1.332364304	0.762622597	0.7773669	0.687737512
LPG226	0.0023093	0.002231636	0.00369729	0.001348884	0.00161321	0.001900147	0.590205	1.057656934	1.022087031	1.693354897	0.617787269	0.738848343	0.870265526
LPE226	0.092794206	0.098245104	0.105239007	0.059729284	0.059445187	0.055676365	0.590157	1.181767738	1.251186901	1.340256779	0.760674012	0.757055941	0.70905863
LPA160	0.075942566	0.067581727	0.077039549	0.043956756	0.042061708	0.044132924	0.590085	1.299217591	1.156181212	1.31798467	0.752007651	0.719587365	0.755021511
LPI202	0.000532936	0.000159395	0.000275935	0.000232585	0.000240784	9.50143E-05	0.587011	2.080901783	0.622373666	1.077416781	0.908149935	0.940165342	0.370992494
LPS151	0.00156924	0.001647217	0.000689838	0.000465173	0.000914914	0.000902606	0.584363	1.521321346	1.596917367	0.668773296	0.45096833	0.88697552	0.875044142
LPC183	0.120384851	0.103000452	0.105067099	0.0669832	0.062286297	0.060945935	0.579126	1.392623679	1.191519256	1.215426425	0.774868182	0.720533946	0.705028512
LPE160	0.045951205	0.044525523	0.049308416	0.03093298	0.024942797	0.024988002	0.578486	1.249529015	1.210761131	1.340820025	0.841145645	0.678257484	0.679486701
LPE203	0.007934748	0.008448229	0.008056993	0.004139833	0.00471906	0.005225646	0.576291	1.23579736	1.315769565	1.254836388	0.644758286	0.734970109	0.813868293
pLPE170	0.000769836	0.001009576	0.00115887	0.000209329	0.000577842	0.00090261	0.575091	0.998045131	1.308853955	1.502403606	0.271382368	0.749136211	1.170178728
LPA201	0.002427786	0.001939362	0.003780079	0.001814078	0.001203902	0.001638902	0.571591	1.137659452	0.908784193	1.771343417	0.850076143	0.56414814	0.767988654
LPC151 p	0.002368599	0.004197546	0.003366205	0.00183739	0.001781663	0.001971433	0.562856	0.915528213	1.622466103	1.301130122	0.710201431	0.688661352	0.762012778
LPS182	0.002901581	0.002975486	0.003642292	0.002186257	0.001252011	0.001876495	0.558311	1.173610623	1.203503523	1.47320832	0.884281495	0.506404633	0.758991406
LPI160	0.006513914	0.00568521	0.006291288	0.0035818	0.002793078	0.003776802	0.549024	1.364547139	1.190948656	1.317911024	0.750322352	0.585099187	0.791171643
LPE150 a	0.001463988	0.000466851	0.001256191	0.000765714	0.000520313	0.000461595	0.548354	1.780050517	0.567640203	1.527391145	0.931025011	0.63264426	0.561248864
LPS226	0.015218277	0.010122226	0.010870953	0.007024035	0.005296975	0.007220521	0.539651	1.637753683	1.089329184	1.169905298	0.755909465	0.570047528	0.777054842
LPI225	0.001065877	0.000850193	0.000662186	0.000651243	0.000433378	0.000285039	0.531235	1.619907891	1.292114511	1.00638287	0.989751744	0.65864367	0.433199314
LPE182	0.084026682	0.081429757	0.083850473	0.044004967	0.042061848	0.037765339	0.496706	1.351131902	1.309373874	1.348298483	0.707590882	0.676345923	0.607258936
pLPE182	0.003552872	0.003241119	0.003200787	0.001907122	0.001300131	0.001520206	0.472993	1.447961341	1.320907583	1.304470369	0.777241222	0.529864358	0.619555128
LPC205	0.768713117	0.690027854	0.717392423	0.339476242	0.353582543	0.325806489	0.4682	1.44359331	1.295827496	1.34721638	0.637514335	0.664005052	0.611843427
LPS203	0.002960853	0.001662276	0.001821152	0.001069898	0.001107589	0.000807614	0.463217	1.88401722	1.057721357	1.15881554	0.68078537	0.704768568	0.513891945
LPE183	0.001480395	0.001062661	0.000717356	0.000558199	0.000337046	0.000570028	0.449413	1.879594103	1.349214889	0.910796502	0.708721519	0.427933375	0.723739612
pLPI200	0.001302713	0.001062693	0.001379635	0.000232574	0.000674144	0.000695617	0.427855	1.461703843	1.192390205	1.548013166	0.260958737	0.756420526	0.780513524
LPS170	0.002901486	0.002284664	0.002897208	0.001209389	0.000818626	0.001282624	0.409562	1.527902575	1.203088327	1.525649768	0.636855856	0.431082675	0.675420799
LPE225	0.008763883	0.009032729	0.008746692	0.004558633	0.002768823	0.00332531	0.401335	1.413678854	1.45704573	1.410905834	0.735341034	0.446631508	0.53639704
LPE205	0.022502447	0.020137068	0.021521834	0.008512598	0.009100652	0.008028208	0.39964	1.503457258	1.345419027	1.437939539	0.568752686	0.608042389	0.536389101
LPE201	0.010007134	0.007757488	0.006953091	0.002093202	0.003274444	0.004037906	0.380519	1.759585571	1.364023224	1.222583673	0.368054249	0.575755643	0.70999764
LPC200	0.26077993	0.250485185	0.25914871	0.061256202	0.063997098	0.064369601	0.246131	1.62981221	1.565472517	1.61961748	0.382836617	0.399966563	0.402294613
LPE200	0.003108885	0.00345372	0.002814368	0.000953584	0.000625995	0.000617553	0.234311	1.611641625	1.790403861	1.458964619	0.494336586	0.324514787	0.320138521

Source data of Figure 1B

Target	NC1	NC2	NC3	NC4	NC5	Tumor1	Tumor2	Tumor3
20_2_CE5	1.002667	0.957735	1.046608	0.694269	0.906801	10.42809365	10.71830986	2.210912732
20_3_CE5	1.120667	1.084928	1.166372	0.872757	2.045956	16.79598662	16.83098592	2.329384282
DPA_n-6	0.027567	0.025243	0.03059	0.019928	0.03477	0.347157191	0.405704225	0.054880694
EPA	0.061407	0.0563	0.057628	0.080938	0.100919	0.85819398	0.909859155	0.113916236
DHA	0.995333	1.169856	1.138053	0.954202	1.691176	13.37792642	14.49295775	1.797096613
20_1_CE5	0.436933	0.577352	0.563422	0.552667	0.74421	5.474247492	5.323943662	0.865843484
AA	0.2646	0.312321	0.303068	0.379007	0.632077	2.964548495	3.012676056	0.551643584
DPA_n-3	0.250733	0.303589	0.267611	0.181334	0.286305	2.220735786	2.023943662	0.286167195
20_5_CE5	0.717333	0.668262	0.753982	0.868424	0.688419	4.83277592	4.631690141	0.861671951
16_1_CE5	0.683333	0.667065	0.739233	0.914841	1.605699	3.522408027	3.614084507	0.626898048
18_2_CE5	9.726667	7.874801	9.976401	9.283327	15.47794	29.78595318	30.78873239	8.651760387
18_1_CE5	9.826667	9.585327	9.59292	13.26897	29.32904	30.09364548	31.51408451	6.444184882
18_3_CE5	0.510667	0.496411	0.527316	0.635722	0.997243	1.742474916	1.750704225	0.274653763
14_1_CE5	0.072133	0.085686	0.094749	0.149276	0.344945	0.327759197	0.350492958	0.097897547
17_0_CE5	0.316733	0.324003	0.376696	0.534348	1.145221	0.91638796	0.911267606	0.298181211
18_0_CE5	5.835333	5.382775	7.905605	15.70739	31.70037	19.6722408	19.74647887	6.529284165
16_0_CE5	11.62	10.33892	14.31268	23.98812	48.4375	26.04013378	27.47887324	6.943100284
20_0_CE5	0.053207	0.083573	0.089322	0.286793	0.494118	0.250301003	0.22943662	0.151059569
14_0_CE5	0.670667	0.879984	0.721534	1.150761	2.522978	1.330434783	1.419014085	0.560987819
12_0_CE5	0.583267	0.527512	0.59705	0.48459	1.145221	0.644214047	0.712676056	0.496579343
15_0_CE5	0.354667	0.330064	0.348968	0.523827	0.59954	0.386555184	0.356549296	0.240780911
13_0_CE5	0.084867	0.07504	0.117581	0.090704	0.322335	0.135518395	0.128450704	0.061121308
15_1_CE5	0.095933	0.084968	0.092448	0.124149	0.334375	0.086956522	0.10443662	0.065626564

Tumor4	Tumor5	Tumor6	Tumor7	FC	Normalized_NC1
3.478790424	7.315669947	15.23253726	14.617737	9.92078	0.175367687
4.123897522	9.212717638	17.733884	18.65443425	9.728826	0.146218463
0.052708946	0.160408781	0.318908242	0.375807	8.873468	0.178456467
0.129693406	0.565253596	0.848087628	0.641862046	8.132609	0.166562003
2.551448971	8.289174868	12.88920812	14.31702345	8.130916	0.162142809
1.377572449	3.239212718	4.428084037	4.449541284	6.251449	0.187036508
0.947921042	1.906131718	3.070569222	3.32993544	5.961629	0.179648662
0.45149097	1.414080242	1.896211169	1.940197078	5.667897	0.261126199
0.919781604	3.047691143	4.415514455	3.936459395	4.375968	0.326778464
0.823183536	2.109008327	4.889567247	4.549779137	3.119644	0.331378737
11.33557329	27.54731264	57.96372778	49.6941896	2.944631	0.43534957
12.64174717	28.74337623	51.28389298	49.6432212	2.098519	0.418204849
0.367156657	0.922785768	2.113485365	1.714237173	2.00381	0.508427191
0.100293994	0.1332324	0.293050817	0.338090384	1.569402	0.362538809
0.422931541	0.637925814	0.962650386	0.946143391	1.349511	0.4877517
11.10457791	11.98334595	27.09642665	26.26571526	1.314073	0.370635529
14.5401092	20.91597275	43.58053511	44.29153925	1.207747	0.476738353
0.162116758	0.128766086	0.182438499	0.193849813	0.920665	0.277000272
0.734565309	0.918243755	1.090141857	1.33503228	0.887573	0.603554264
0.614447711	0.328917487	0.558807685	0.421848454	0.808418	0.983706576
0.304913902	0.272445117	0.339378704	0.423207611	0.769508	0.949809956
0.084586308	0.064761544	0.138319267	0.076299694	0.712766	0.738193684
0.080680386	0.051241484	0.045914886	0.069334013	0.492074	0.931343123

Normalized_NC2	Normalized_NC3	Normalized_NC4
0.167509124	0.183053025	0.121428556
0.141555507	0.152181804	0.113872503
0.163415338	0.198028225	0.129007899
0.152710035	0.156313454	0.219540243
0.190573154	0.185392291	0.155442432
0.247145233	0.241181992	0.236578385
0.212048274	0.205766187	0.257324848
0.316172223	0.278703046	0.188850609
0.304424006	0.343473761	0.395607382
0.323489688	0.358486991	0.443647078
0.352463099	0.446527276	0.415506424
0.407933875	0.408257036	0.564702899
0.494234523	0.525003147	0.632934349
0.430652909	0.476205427	0.750253797
0.498946874	0.580091123	0.82286665
0.341890958	0.502130353	0.997666508
0.42417879	0.587212184	0.984170014
0.435088786	0.465018592	1.493078253
0.791925636	0.649331326	1.035606634
0.889673653	1.006953063	0.817283207
0.883922592	0.934547523	1.402827902
0.652717516	1.022753034	0.788970987
0.824890106	0.897510361	1.205267655

Normalized_NC5	Normalized_Tumor1	Normalized_Tumor2
0.158600741	1.823886967	1.874646155
0.266945144	2.19144856	2.196015076
0.225089627	2.247368043	2.626380025
0.273737223	2.327801136	2.467939911
0.27549776	2.179304663	2.360946637
0.318571157	2.343341784	2.279001755
0.429145216	2.012763302	2.045439236
0.29817246	2.312785007	2.107835874
0.313606703	2.201552635	2.109948776
0.778674659	1.708172374	1.752630378
0.692767139	1.333170177	1.378052922
1.248190144	1.280730171	1.341181441
0.992869344	1.734833475	1.743026695
1.733676935	1.647302622	1.761561454
1.763575949	1.411186441	1.403301381
2.013472387	1.24949698	1.254212266
1.987264544	1.068358907	1.127386642
2.572435586	1.303096969	1.194474492
2.270508093	1.197300576	1.277015907
1.931468209	1.08649719	1.201961579
1.605590639	1.03520854	0.954851704
2.803754935	1.178776391	1.117299669
3.246190306	0.844194147	1.013895006

Normalized_Tumor3	Normalized_Tumor4	Normalized_Tumor5
0.386691475	0.608444911	1.279520066
0.303925333	0.538063615	1.202025058
0.355277441	0.34121834	1.038427486
0.308991148	0.351785803	1.533217423
0.292752472	0.415638752	1.350331649
0.370638561	0.589692571	1.386598345
0.374535267	0.643585588	1.294157265
0.29802879	0.470205214	1.472693691
0.39253137	0.419003001	1.388364075
0.304010756	0.399198322	1.022751973
0.387238537	0.507361579	1.232972316
0.274252649	0.538009495	1.223265196
0.273449297	0.36554653	0.918738988
0.492028561	0.504072993	0.669619901
0.459182465	0.651291028	0.982370239
0.414712331	0.70531551	0.761131115
0.284857332	0.596542833	0.858127918
0.786434191	0.843999236	0.670371651
0.504850781	0.661058684	0.82635676
0.837504341	1.03629487	0.554734761
0.644819849	0.816570281	0.729617721
0.531650004	0.735755049	0.563313782
0.637117957	0.783263965	0.497464247

Normalized_Tumor6	Normalized_Tumor7
2.664190323	2.556660969
2.313820285	2.433928651
2.064494734	2.432836375
2.300388245	1.741013376
2.099690973	2.332286408
1.89551429	1.904699418
2.084745471	2.260840686
1.974808885	2.020618004
2.011470767	1.793239061
2.371168709	2.206390336
2.594360931	2.22423003
2.182548108	2.112724136
2.104216896	1.706719565
1.472859902	1.69922669
1.482428002	1.457008149
1.721049656	1.668286405
1.787995917	1.817166566
0.949796652	1.009205319
0.981053329	1.20143801
0.942455358	0.711467194
0.908868247	1.133365045
1.203139154	0.663675794
0.445752397	0.673110731

Source data of Figure 1C

Target	Healthy1_1	Healthy1_2	Healthy1_3	Healthy2_1	Healthy2_2	Healthy2_3	Healthy3_1
AA	0.353178582	0.298290897	0.324139352	0.24677512	0.242016029	0.233535283	0.453823713
20_3_CE5	1.846504349	1.757307631	2.017590023	1.161576758	1.266586564	1.04279281	2.503164294
EPA	0.107376445	0.090701383	0.120350935	0.205492594	0.191983681	0.198843329	0.867846891
20_5_CE5	1.288468131	1.110780483	1.146098973	1.274506557	1.470475904	1.228529166	4.997292825
rLN_1	0.037836632	0.035615885	0.053933334	0.022995255	0.021794943	0.022663885	0.045042327
rLN_2	0.011248784	0.01852033	0.020974053	0.011814099	0.013447879	0.007269456	0.025985992
aLN_rLN	0.101239213	0.091414005	0.098128562	0.054851551	0.060283945	0.062430757	0.131085123
16_1_CE5	10.05791182	9.300858095	11.16114909	4.902477738	5.341339999	4.946493768	13.74047549
18_3_CE5	9.847850769	9.655677724	10.64308617	5.75978759	6.7290872	6.306359381	13.36347194
20_2_CE5	1.236279822	1.21141023	1.262777888	0.723020513	0.754007625	0.760763167	1.865642672
14_1_CE5	0.603831051	0.564167127	0.641198182	0.678285172	0.761124136	0.731280612	0.475806161
20_0_CE5	0.205034983	0.236252726	0.257190443	0.34514235	0.355187048	0.30277525	0.48996729
15_0_CE5	0.779973702	0.719457985	0.712374055	1.103576583	1.104099305	1.024182703	1.005605191
14_0_CE5	3.184239708	2.958028043	2.904558964	7.033866011	7.366463384	6.545121315	5.349760798
18_2_CE5	54.69916245	52.38636757	51.5044099	37.28553748	40.02261975	36.46904606	66.71111039
DPA n-3	0.16415897	0.169659821	0.17994521	0.18658442	0.139937168	0.142021073	0.70367466
16_0_CE5	41.93548635	42.18160196	47.85315433	41.03183833	44.34870341	42.68101034	59.56620076
20_1_CE5	1.886742995	1.702939537	1.709159805	1.800363623	1.979628167	1.704319914	5.374987823
17_0_CE5	1.594178676	1.457399174	1.44629696	1.739078568	1.796889125	1.674419678	2.991026051
18_1_CE5	100.0689808	84.9367041	85.45841621	70.30105048	73.17699331	68.76819854	111.4534426
15_1_CE5	0.238777514	0.236967485	0.240706555	0.152532888	0.204034253	0.152662389	0.268223301
DPA n-6	0.048698699	0.023387037	0.031663095	0.035806355	0.04298645	0.035671984	0.160744751
13_0_CE5	0.193782416	0.158610756	0.188523626	0.220669677	0.301874823	0.245673883	0.453866212
18_0_CE5	30.24831524	28.13714392	30.95109132	34.1319738	37.03156134	33.74612094	47.24030607
DHA	1.886112815	1.734085404	1.796884087	2.184404999	2.350008411	2.048276657	10.51293642
12_0_CE5	1.786259607	1.650214695	1.641153371	9.701515703	10.45820051	10.3426311	0.174680116

Healthy3_2	Healthy3_3	Healthy4_1	Healthy4_2	Healthy4_3	Healthy5_1	Healthy5_2	Healthy5_3	Patient1_1
0.452210597	0.469468347	0.577986324	0.594214112	0.611675851	0.351066222	0.409505815	0.467438557	0.5484868
2.202411274	2.259460827	3.290122656	3.460232188	3.62823634	1.814586189	1.898310262	1.994438009	4.9342725
0.840078276	0.851724592	0.601972653	0.662532453	0.611912702	0.331294763	0.309473287	0.310506617	0.282974
4.602128576	4.882844506	3.281289363	3.487703903	3.302220635	1.963048509	2.017517436	2.021084636	2.2498095
0.043078673	0.045502626	0.063655686	0.062111773	0.078766375	0.044003167	0.047304853	0.046807645	0.0648774
0.024232056	0.024089383	0.03759194	0.031449184	0.025719647	0.026655179	0.032715825	0.02734308	0.0304425
0.136776161	0.136506368	0.224048156	0.211494538	0.223439609	0.144065805	0.137052596	0.156643405	0.1831547
11.92162078	11.99999324	21.61002607	22.17856433	23.09611345	22.63430836	26.29829383	27.89557129	29.477879
11.94901999	11.91833772	21.6469545	21.43616556	22.50415178	16.64567145	15.75772159	17.10101838	16.490885
1.722797714	1.819657909	2.236473086	2.39076161	2.371200035	1.149006508	1.213088352	1.259171983	2.2143144
0.299426773	0.456662424	1.783896747	1.986940964	1.804609082	1.812287532	2.081733223	2.066991165	0.1968806
0.434583703	0.412765866	0.431557998	0.415623734	0.392243037	0.295778266	0.320061132	0.347118325	0.5235155
0.928174402	1.111386477	0.756377556	0.980121267	0.895105824	1.455551737	1.55611218	1.633013568	1.3300369
5.091399472	4.962733446	6.8839017	7.419891556	7.493057298	9.099594952	11.33395321	12.22309129	6.5097813
61.25071507	62.07941821	76.21401047	84.76779306	83.00615555	77.17366552	65.20353597	71.80264238	77.434638
0.629520493	0.457664068	0.421279287	0.383440111	0.429910286	0.218946541	0.260055668	0.304957479	0.3110483
55.24138116	56.01582682	69.56973413	73.85957075	78.74864939	56.26885904	71.18598739	73.15564686	76.424286
4.886668356	4.661777286	3.194348412	3.236326817	3.375487411	2.574069218	3.073948143	3.182609798	3.3831211
2.485882575	2.679733081	2.366039496	2.643921118	2.610708145	2.326383821	2.461942973	2.562371702	2.2208029
101.9271868	96.66575338	108.9857869	115.7284466	115.3944711	102.3145121	115.4956999	126.4219996	106.19877
0.192517218	0.150430617	0.224047964	0.256304091	0.275419182	0.236946413	0.255529005	0.31468166	0.0848396
0.145122764	0.16620189	0.112476778	0.121024251	0.109358641	0.052456963	0.05998577	0.066595711	0.0473603
0.424362096	0.426681377	0.206577389	0.202317312	0.229880586	0.233563312	0.209330532	0.306343698	0.0948219
42.2987997	43.02871969	46.32195782	47.89608163	49.0052323	33.64327868	33.06867517	33.23569594	44.547659
9.106470125	9.059728278	5.468390692	5.586043954	5.764194759	2.778630382	3.497686324	3.552519071	2.607325
0.197632437	0.671327382	3.171386372	3.087260257	3.29801689	5.037624456	5.117105059	5.603675336	0.3124165

Patient1_2	Patient1_3	Patient2_1	Patient2_2	Patient2_3	Patient3_1
0.504430984	0.596689506	0.520004225	0.520435695	0.588556051	0.795768066
4.680596039	4.7810941	3.084228708	3.055668221	3.093799205	2.678982658
0.259424408	0.281902094	0.304861334	0.301591553	0.308930562	0.344209909
2.171725755	2.330038648	2.42951132	2.368396519	2.590240652	2.373006394
0.068747686	0.064284053	0.043445577	0.050304561	0.041364339	0.022016609
0.022079715	0.027399943	0.026509287	0.024069312	0.029845557	0.013961843
0.155058445	0.182049877	0.119783201	0.098203002	0.116502402	0.06068046
27.23798086	29.9350914	28.26216008	26.14344045	29.60593146	15.74063893
15.84557821	16.71205916	13.6208815	12.59694198	13.87082575	5.532118006
1.896190944	2.096666885	1.834677951	1.624315873	1.696607711	0.843631609
0.040142421	0.13436003	1.448112483	1.334083788	1.501088995	0.03705117
0.441830365	0.593821231	0.354417777	0.365591139	0.347401298	0.477911348
1.295107623	1.394201704	2.165608421	2.036659391	2.461897282	1.095988582
6.329013288	6.844764471	11.75750579	12.00950897	12.27443289	4.192117937
74.92871399	74.48321722	67.55638261	64.99792848	69.85214996	38.98916202
0.328486698	0.358030574	0.519934227	0.522084168	0.541595615	0.185693729
69.63979647	77.32975091	72.78798358	70.18151021	75.3057159	49.78473829
3.025409265	3.15877332	3.010007189	2.755442425	3.071967362	1.807189041
2.334485678	2.483409609	2.773502978	2.645960027	2.834548596	1.398359365
106.8474804	116.7432269	100.7126721	107.4382499	112.8337381	87.96571792
0.060719638	0.073241318	0.19783654	0.24020945	0.218342794	0.121360879
0.043316076	0.054138971	0.098525842	0.044079714	0.064043623	0.065275433
0.035125188	0.106434714	0.279323406	0.291955546	0.336143284	0.23976179
40.10050155	46.08105808	32.83749464	30.24621925	33.03707236	28.79329759
2.647877799	2.687232916	3.598669599	3.729549513	4.03098342	3.110183662
0.124943563	0.144903824	2.521652356	2.35380964	2.338237306	0.139080594

Patient3_2	Patient3_3	Patient4_1	Patient4_2	Patient4_3	Patient5_1
0.801598993	0.789641381	0.78635722	0.768229566	0.799158779	3.135242715
2.621141723	2.520515538	3.87318995	3.648669159	4.182129086	10.90647886
0.358546082	0.316905067	0.208927879	0.243097473	0.220127534	1.412536054
2.764609047	2.693432834	1.935648528	1.898871176	2.0124619	8.107451512
0.024987892	0.03035316	0.065799116	0.0745957	0.08707537	0.252802202
0.009777864	0.012346789	0.022530416	0.023995848	0.027042441	0.125315562
0.06491517	0.069966038	0.118796922	0.136150301	0.125205017	0.670762902
15.60143137	15.37701794	10.91229598	11.93554544	13.1093503	39.62050225
5.42353025	5.21558764	9.168347136	9.115017869	9.913099117	58.25469874
0.900692964	0.73337782	1.581323086	1.537916423	1.826306826	3.060179848
0.157542642	0.23587577	0.717922339	0.688855794	0.781211277	3.482948092
0.431891619	0.349307824	0.362791342	0.372978977	0.411019631	0.900804804
0.996855242	0.936297784	0.729939654	0.927496449	0.889123276	2.530400452
4.132547869	3.990779455	3.463529324	3.325737833	3.566033123	18.90102937
37.25553634	38.28926214	51.78507236	49.71795493	54.90435852	141.0074479
0.197889911	0.194280893	0.224901988	0.209325963	0.177289063	0.803658057
51.27116228	47.40679793	39.18506898	37.80052029	47.4775653	94.54067972
1.831786759	1.765568486	1.591470746	1.458021494	1.599283138	4.272764137
1.367335837	1.226236102	1.294801507	1.304708919	1.169764124	4.378189592
91.35670051	79.48584116	72.10683164	70.22338819	69.65989182	141.0400589
0.101040727	0.038838963	0.245793659	0.22587898	0.229857404	0.504156822
0.056780288	0.048168892	0.011899226	0.008107702	0.025833017	0.187270513
0.207788737	0.269830517	0.292383935	0.272560888	0.294481211	0.337783474
28.02270213	25.57430647	30.98122808	29.11608021	30.41106597	66.0425916
3.098833967	3.077422447	0.879231109	0.861283269	0.918843388	8.078178826
0.126032887	0.217094424	2.193756323	2.132075775	2.090911138	12.56631385

Patient5_2	Patient5_3	Patient6_1	Patient6_2	Patient6_3	Patient7_1
3.328570591	3.413188765	0.660725092	0.520211498	0.536389042	1.216948667
11.23942562	11.3866683	2.463356726	1.959507204	1.922998897	2.738507952
1.536881396	1.509974401	0.148731735	0.149003217	0.16729759	1.194194301
8.51089514	8.105450585	1.760851055	1.668562414	1.546921526	5.878279266
0.24371699	0.232797979	0.030115075	0.032546788	0.016173818	0.02949265
0.12957099	0.12949702	0.013521054	0.007628135	0.007581293	0.016251133
0.684876	0.73101557	0.06944905	0.059245202	0.054586189	0.051763798
41.2460223	42.19726744	7.903766635	6.028140967	5.899659961	7.144792668
59.32798183	61.12456742	7.311204784	5.953518128	5.864938159	6.326586535
3.55343571	3.202332393	1.179097114	1.14582394	1.0129285	0.533250103
3.809311631	3.456273005	0.753827052	0.680156436	0.704108543	0.600668858
0.878156693	0.933669077	0.505505457	0.412950773	0.370000146	0.322014198
2.752781288	2.43878382	1.177598248	1.195088237	1.00836637	0.735202841
20.60279629	19.32713432	4.686129194	3.976901241	4.051042777	3.34813135
146.9891956	144.0902185	44.2697102	41.41479283	40.2352937	47.52247852
0.936489294	0.839728856	0.157012067	0.137925235	0.115377702	0.129254831
94.8973382	98.65515963	51.66187987	45.01172186	43.31074112	36.93471396
4.386385009	4.057928438	1.725202203	1.452675736	1.383148965	0.747259763
4.51430699	3.929150816	2.245109939	1.932827446	1.95434517	1.170624483
159.1125483	140.5664318	85.1182934	70.42525957	70.90551289	59.80604811
0.497715359	0.567414092	0.25382706	0.242576753	0.257775991	0.217284611
0.088986773	0.140730544	0.028844035	0.137925235	0.115377702	0.129254831
0.353738368	0.341539604	0.255674121	0.167573759	0.152139206	0.217882623
67.40650539	64.59991998	34.26660729	31.09320441	28.77386249	23.2048394
8.672545446	8.282652688	1.999895389	1.633510306	1.576085772	3.206756139
14.04763047	13.42418712	3.307131728	2.384968948	2.393508154	2.517173366

Patient7_2	Patient7_3	Patient8_1	Patient8_2	Patient8_3	Patient9_1	Patient9_2
1.212669031	1.22331287	1.47712855	1.536858349	1.757026239	0.214239257	0.212942404
2.690605693	2.901747894	5.37448774	5.548217527	5.73307397	2.003478023	2.007984732
1.124510041	1.190354951	0.464973348	0.490255938	0.554410528	0.449870993	0.35522542
6.140931946	6.323274914	3.022061684	3.275486935	3.537911116	3.02058	2.83742863
0.027150281	0.037029586	0.076833259	0.0686304	0.089015373	0.024655992	0.018408718
0.009615931	0.010153249	0.033559325	0.036145441	0.026607812	0.01291456	0.007138312
0.054301123	0.062114174	0.187226011	0.204518535	0.209476195	0.055181894	0.052409506
6.155516906	7.23135614	40.9996663	42.43117179	45.34304499	7.105011586	6.980213304
5.973165458	6.436276732	18.60953661	18.65600494	18.79046261	6.695687429	6.01302061
0.507351254	0.514260884	2.608579464	2.802420718	2.758446293	0.464959392	0.337724037
0.537329012	0.584126689	2.715685684	2.841608234	2.955452031	0.779626575	0.666832971
0.303165743	0.289071014	0.361426127	0.32301564	0.352208351	0.181212647	0.160418577
0.697410469	0.758512735	1.083206872	1.179027916	1.444583819	0.992135897	0.890741001
3.011862371	3.081397529	7.761138483	8.547460349	9.647896152	3.642958812	3.493568475
44.08564893	45.23036692	86.71441791	91.60904454	96.44812395	13.35379186	13.38886405
0.159196513	0.152802613	0.33165534	0.314296288	0.413408377	0.098650644	0.096680521
33.32741884	38.14177437	65.99236093	70.41690659	74.29236693	35.70364417	34.60102006
0.755686548	0.906619009	4.787175369	4.891952212	4.942826471	0.56514207	0.630038715
1.080319232	1.057179378	2.163697942	2.1861484	2.470797397	1.900147332	1.672661756
55.18084782	62.42135526	116.0789376	126.0399572	144.5204935	58.66854287	57.41120482
0.26924133	0.206651982	0.245512957	0.258732108	0.280352222	0.147935058	0.14539284
0.159196513	0.152802613	0.083552646	0.1215238	0.090130048	0.006815299	0.006248851
0.23586488	0.301911318	0.460120455	0.512662563	0.515252446	0.158115901	0.122097071
21.10384638	22.3840169	34.66464953	37.19618203	38.14486084	26.32755941	26.23737289
3.107449999	3.227153077	4.462521477	4.655604326	5.026398295	0.497860029	0.484618941
2.382489126	2.474544011	3.939782237	4.107789851	4.399541555	1.528663308	1.649510571

Patient9_3	Patient10_1	Patient10_2	Patient10_3
0.251876984	0.978454024	0.964258782	1.016980953
2.29146958	1.441574576	1.344530023	1.483402531
0.446873481	2.071283835	1.94083411	2.00877034
3.075937978	10.36776508	10.3228615	10.58949847
0.027355334	0.013967381	0.012907258	0.02229059
0.012946855	0.007565664	0.010662729	0.015838057
0.0630635	0.064599135	0.051068305	0.046340885
7.914944992	3.620773099	3.378149241	3.459203179
6.830905347	6.976126194	6.664423991	6.624394148
0.493262852	0.432988755	0.433206632	0.417041524
0.731285705	0.06110729	0.143098845	0.294238513
0.200474924	0.316015315	0.331374204	0.27861182
0.938027022	0.953891922	0.936020521	0.859605027
3.789606394	3.040484386	3.065302189	3.000384513
14.78493284	33.02122795	31.1211905	32.243509
0.106885084	0.140011228	0.098874414	0.15132955
38.46642444	23.12470284	22.33972666	23.57613557
0.671574493	2.006096467	1.827782079	1.976197157
1.842736014	1.000996276	0.973872765	1.066366196
61.06889902	39.66204681	37.72307034	40.37048057
0.13281291	0.109702069	0.085301834	0.104998628
0.017806055	0.088092778	0.099515603	0.076110411
0.166644427	0.246178324	0.248596084	0.34314127
29.28243502	17.00267116	15.95583725	16.67374434
0.488872344	5.104501764	4.714252546	5.088938202
1.692224389	0.448712311	0.139730255	0.072733232

Patient11_1	Patient11_2	Patient11_3	Patient12_1	Patient12_2	Patient12_3
0.897658898	0.895787303	1.00823281	2.20279902	2.042711855	2.08346923
10.05291636	9.140111223	9.011767217	8.044693218	7.33904269	7.56585043
0.510982626	0.453932388	0.470648156	2.695705712	2.591936691	2.626516447
2.410497737	2.597750638	2.764875147	13.45992594	12.48821316	12.80584662
0.251982202	0.236884516	0.219224466	0.093718939	0.094235184	0.087501562
0.118184073	0.115960969	0.12396764	0.037847737	0.036678475	0.044326414
0.859054347	0.791616142	0.698726983	0.231292719	0.221762953	0.217026965
60.34165925	56.59720021	56.11357688	22.27578143	20.20177355	21.27712464
71.54146058	65.86466078	63.79028673	22.67871951	21.26385878	22.35034382
5.436988444	5.220658802	4.574738358	2.030096726	2.075812563	1.748743705
3.622102122	3.483433266	3.486304445	2.125066594	1.870914927	1.926669643
0.625114093	0.692678797	0.690441492	0.328643332	0.355500201	0.34942491
1.077388593	1.07796698	1.081645051	1.273986599	1.235503197	1.231591969
14.7837259	13.86698697	15.01739138	10.05227992	9.027690187	9.543999975
147.5488473	141.5214378	134.7733244	104.2624136	97.2247772	101.4347891
0.632344343	0.645931322	0.708317715	0.522245928	0.378970229	0.371422861
102.1985489	94.24831547	105.0878277	75.82441827	72.96334574	71.65261217
9.084406458	8.059235203	8.315322057	4.230167734	3.821272459	4.115143565
3.761548659	3.810376802	3.77398296	2.831583346	2.493668965	2.564972739
168.7516808	170.3538021	202.8308465	137.9857209	128.0664445	132.0115074
0.34136819	0.375061778	0.343730154	0.293179777	0.296256035	0.205800444
0.0943445	0.081849201	0.057683705	0.12731771	0.10391816	0.139294677
0.180373406	0.169775652	0.165298946	0.287780244	0.31600059	0.370145436
59.94112788	56.59733567	54.47704023	42.89694154	38.38842674	40.39612389
3.856928138	3.544512643	3.613419124	7.941203073	7.088696391	7.602957006
4.549891924	4.196245575	4.491082619	3.314601359	3.308977431	3.501342712

FC	Ave	Normalized_Healthy1	Normalized_Healthy1	Normalized_Healthy1	Normalized_Healthy	Normalized_Healthy
2.794088	0.919458	0.384116	0.324420313	0.352533018	0.268391904	0.26321593
2.200382	3.958618	0.466451796	0.443919507	0.509670335	0.293429889	0.319956776
1.983284	0.711751	0.15086241	0.127434179	0.169091388	0.288714232	0.269734398
1.887082	4.127667	0.312154113	0.269106148	0.277662675	0.308771675	0.356248703
1.78639	0.069576	0.54381345	0.511895394	0.775166061	0.330503232	0.313251546
1.670664	0.033305	0.33775423	0.556088521	0.629763637	0.354728286	0.403783908
1.654841	0.191988	0.5273197	0.476143622	0.511117402	0.28570257	0.31399801
1.495043	20.42922	0.492329675	0.455272279	0.546332579	0.239973795	0.26145588
1.476971	17.93514	0.54908139	0.538366499	0.593420908	0.32114542	0.375190144
1.238665	1.71189	0.722172303	0.707644742	0.737651137	0.422352108	0.440453216
1.233595	1.300658	0.464250541	0.433755259	0.492979952	0.52149398	0.585184037
1.231758	0.406582	0.504289946	0.58107096	0.632567929	0.848888393	0.873593641
1.210708	1.207329	0.646032309	0.595908685	0.590041247	0.914064315	0.914497272
1.158671	7.40221	0.43017419	0.399614173	0.392390779	0.950238642	0.995170813
1.147148	67.7464	0.807410612	0.773271604	0.760253087	0.550369279	0.590771164
1.061983	0.333427	0.492338319	0.508836227	0.539683711	0.559595735	0.419693363
1.055068	59.12173	0.709307474	0.713470336	0.809400414	0.694022941	0.75012524
1.015466	2.988498	0.631334778	0.569831163	0.571912565	0.602430841	0.662415662
1.012974	2.209133	0.721630826	0.659715367	0.65468977	0.787222112	0.813390998
1.012033	99.30961	1.007646493	0.855271747	0.860525137	0.707897756	0.736857117
1.007509	0.227853	1.04794378	1.03999995	1.056409933	0.66943444	0.895462988
0.997555	0.080673	0.6036585	0.289900636	0.392488851	0.443847803	0.532850705
0.973135	0.261744	0.740350108	0.605975984	0.720258783	0.843073497	1.153319596
0.945059	36.52532	0.828146472	0.770346258	0.847387264	0.934474316	1.013859999
0.836738	3.971163	0.474952236	0.436669394	0.452483069	0.550066799	0.591768287
0.750296	3.401419	0.525151285	0.485154769	0.482490786	2.85219652	3.074658023

Normalized_Healthy	Normalized_Hea	Normalized_Hea	Normalized_Hea	Normalized_Hea	Normalized_Hea	Normalized_Hea	Normalized_Healt
0.253992295	0.493577353	0.491822932	0.510592411	0.628616248	0.646265543	0.66525688	0.381818603
0.263423468	0.632332917	0.556358665	0.570770148	0.831129168	0.87410112	0.916541225	0.45838884
0.279372109	1.219312796	1.180298278	1.19666119	0.845763194	0.93084887	0.859728825	0.465464528
0.297632842	1.210682259	1.114946755	1.182955137	0.794950177	0.844957738	0.800021147	0.475583098
0.325740565	0.647378542	0.619155585	0.65399427	0.914902215	0.892712065	1.132083182	0.632443034
0.218271542	0.780251319	0.727587928	0.723304041	1.128729707	0.944288272	0.772254086	0.800344237
0.325180009	0.682776615	0.712419245	0.711013991	1.166988583	1.101601174	1.163818875	0.750388459
0.242128357	0.672589296	0.583557281	0.587393574	1.057799799	1.085629458	1.130543021	1.107937897
0.351620334	0.745100014	0.666235167	0.664524432	1.206957756	1.195204909	1.25475205	0.92810387
0.444399463	1.08981433	1.006371512	1.062952351	1.30643475	1.396562322	1.385135437	0.671191636
0.562239088	0.365819656	0.230211813	0.351101152	1.371534353	1.527643231	1.387458862	1.393362375
0.744685187	1.205089858	1.068872196	1.015210542	1.061430379	1.022239559	0.964734003	0.727475885
0.848304391	0.832917112	0.768783166	0.920533051	0.626488223	0.811809428	0.741393307	1.205596351
0.88421178	0.722724804	0.687821535	0.670439426	0.929979245	1.002388681	1.012272991	1.229307856
0.538317105	0.984718157	0.904117634	0.916350064	1.124989818	1.25125162	1.225248209	1.139155221
0.425943319	2.110429903	1.888030005	1.372605821	1.263482196	1.149996614	1.289367907	0.656654777
0.721917456	1.007517858	0.93436676	0.947465931	1.176720164	1.24927955	1.331974669	0.951745782
0.570293059	1.798558021	1.635158414	1.559906218	1.068880739	1.082927394	1.129492783	0.86132527
0.757953217	1.353936439	1.125275054	1.213024629	1.071026142	1.196813763	1.181779373	1.053075357
0.692462675	1.122282546	1.026357734	0.97337763	1.09743444	1.16532978	1.161966807	1.030257913
0.670002793	1.177175083	0.844917169	0.660208021	0.983298917	1.124864203	1.208756279	1.039907468
0.442182168	1.992557041	1.798910277	2.060202545	1.394237728	1.500190341	1.35558597	0.650245122
0.938602632	1.73400614	1.621284995	1.630145861	0.789233593	0.772957872	0.878263982	0.892333921
0.923910333	1.293357745	1.158067861	1.178051806	1.268214961	1.311311744	1.341678368	0.92109469
0.515787587	2.647319193	2.293149334	2.281379018	1.37702494	1.406651842	1.451512957	0.699701896
3.040681204	0.05135507	0.058102936	0.197366853	0.932371544	0.907638892	0.969600276	1.481036096

Normalized_Healt	Normalized_Healt	Normalized_P	Normalized_P	Normalized_P	Normalized_Patient2_1	Normalized_Patient2_2
0.445377335	0.508384817	0.596532661	0.548617673	0.648957774	0.565555085	0.566024351
0.479538665	0.503821826	1.246463499	1.182381418	1.207768577	0.779117591	0.771902829
0.434805659	0.436257472	0.397574533	0.36448768	0.396068516	0.428325929	0.423731932
0.488779156	0.489643373	0.545056002	0.526138839	0.564492927	0.588591935	0.573785797
0.679897084	0.672750867	0.932461094	0.988087861	0.923933525	0.624428973	0.723010897
0.98232022	0.820999018	0.914061096	0.66296207	0.8227064	0.795963705	0.722701384
0.713859098	0.815900779	0.953988609	0.807645276	0.948234218	0.623908852	0.511505137
1.287288125	1.365474045	1.442927221	1.333285327	1.465307518	1.38341838	1.27970813
0.878594922	0.953492408	0.919473505	0.883493496	0.931805415	0.759452263	0.70236101
0.708625017	0.735544749	1.293490749	1.107659089	1.22476702	1.071726302	0.948843388
1.60052348	1.589189169	0.151370022	0.030863171	0.103301605	1.113369381	1.025699356
0.787200354	0.853748364	1.287602725	1.086695585	1.46052187	0.871701597	0.899182828
1.288888	1.352583456	1.101635614	1.072704587	1.154781685	1.793718179	1.686912988
1.531158013	1.651275933	0.879437524	0.855016712	0.924692009	1.588377759	1.622422076
0.962464954	1.059873914	1.143007422	1.106017644	1.099441697	0.997195163	0.959429996
0.779947452	0.914614976	0.93288241	0.98518277	1.073789454	1.559363725	1.565811734
1.204057882	1.237373203	1.292659791	1.177905216	1.30797506	1.231154453	1.1870679
1.028592858	1.064952809	1.132047156	1.012350963	1.056976737	1.007197179	0.922015684
1.114438405	1.159899098	1.005282462	1.056742794	1.124155626	1.255471094	1.197736709
1.162986132	1.273008714	1.069370478	1.075902723	1.175548129	1.014128154	1.081851488
1.121462516	1.381070951	0.372342932	0.266485592	0.321440586	0.868262543	1.054228242
0.743570587	0.825505972	0.587068293	0.536936672	0.671094937	1.221304949	0.546402568
0.799752038	1.170393033	0.362269205	0.134196576	0.406636234	1.067161396	1.115422773
0.905363041	0.90993578	1.21963774	1.097882266	1.261619544	0.899033719	0.828089087
0.880771249	0.894578979	0.656564575	0.666776383	0.676686607	0.906200392	0.939157969
1.504402991	1.64745219	0.091848882	0.036732775	0.042600991	0.741353031	0.692008122

Normalized_Patient2_3	Normalized_Patient3_1	Normalized_Patient3_2	Normalized_Patient3_3	Normalized_Patient	Normalized_Patient
0.640111852	0.865475038	0.871816737	0.858811673	0.855239829	0.835524245
0.781535228	0.676746997	0.6621356	0.636716074	0.978419796	0.921702829
0.434043137	0.483610128	0.503752252	0.445247206	0.293540761	0.341548564
0.627531448	0.574902621	0.669775265	0.652531573	0.46894497	0.460035008
0.594516029	0.316437469	0.359142746	0.436255971	0.94570904	1.072139437
0.896138012	0.419216102	0.293588599	0.370722739	0.676494727	0.720495567
0.606820319	0.31606332	0.338120443	0.364428653	0.618771669	0.70915936
1.449195306	0.77049628	0.763682141	0.752697217	0.534151345	0.584238887
0.773388271	0.308451368	0.302396898	0.290802753	0.511194666	0.508221215
0.991072633	0.492807026	0.526139391	0.428402325	0.923729171	0.898373188
1.154099938	0.028486487	0.121125365	0.181351147	0.55196869	0.529621116
0.854444346	1.175437892	1.06225093	0.859133507	0.892296641	0.917353446
2.039126679	0.907779368	0.825669752	0.775511556	0.604590384	0.768221634
1.658211916	0.566333286	0.558285681	0.539133507	0.467904762	0.449289849
1.031082828	0.575516365	0.549926435	0.565185191	0.764395926	0.733883347
1.624329602	0.556924416	0.593502664	0.582678656	0.674516091	0.627801165
1.273740017	0.842071716	0.867213469	0.801850628	0.662786215	0.639367607
1.027930057	0.604714736	0.612945531	0.590787824	0.532531899	0.487877617
1.283104382	0.63299004	0.618946737	0.555075654	0.586112897	0.590597647
1.136181458	0.885772459	0.919918022	0.80038418	0.726081104	0.707115734
0.958260134	0.532627115	0.443446286	0.170455956	1.078736151	0.991334854
0.793870845	0.809140095	0.703836126	0.597091132	0.147500217	0.100501319
1.284243026	0.916015346	0.793861574	1.030893601	1.117059443	1.041325044
0.904497811	0.788310609	0.767213041	0.700180208	0.848212356	0.797147838
1.015063666	0.783192115	0.780334087	0.774942337	0.221403925	0.216884383
0.687429935	0.040888991	0.037053031	0.063824662	0.644953257	0.626819488

Normalized_Patient	Normalized_Patient5	Normalized_Patient5	Normalized_Patient5	Normalized_Patient6_1	Normalized_Patient6_2
0.869162767	3.409880872	3.620143709	3.712174189	0.718602691	0.565780514
1.056461971	2.755123029	2.839229853	2.876425329	0.622276991	0.494997835
0.30927612	1.984593484	2.159296957	2.121493004	0.208966016	0.209347445
0.487554363	1.964173014	2.061914344	1.963688254	0.426597201	0.404238652
1.251505632	3.633442828	3.502864066	3.345928724	0.432834058	0.467784263
0.811972083	3.762705415	3.890478228	3.888257209	0.405981049	0.22904119
0.652149199	3.493769658	3.567279857	3.807604761	0.361735845	0.308587565
0.641696038	1.93940346	2.01897184	2.065534807	0.386885362	0.295074438
0.552719407	3.248076327	3.307918803	3.408090072	0.407646966	0.331947151
1.066836313	1.787602685	2.075737876	1.870641031	0.68876905	0.66933254
0.60062787	2.677836016	2.92875794	2.657327095	0.579573734	0.522932686
1.010915623	2.215557559	2.159853823	2.296388265	1.243306465	1.015665327
0.736438115	2.095866107	2.280058477	2.019982389	0.975374571	0.989861083
0.481752491	2.553430567	2.783330409	2.610995125	0.633071633	0.537258632
0.810439497	2.081401335	2.169697506	2.126905902	0.653462177	0.611320936
0.531717512	2.410295683	2.80867725	2.518477626	0.470903645	0.413659262
0.803047606	1.599085084	1.605117696	1.668678442	0.873822166	0.761339703
0.535146052	1.429736115	1.4677555	1.357848609	0.577280612	0.48608884
0.529512698	1.981858508	2.043474255	1.778593825	1.016285417	0.874925683
0.701441597	1.420205539	1.602186812	1.415436341	0.857100264	0.709148482
1.008795314	2.212637184	2.18436697	2.490259901	1.113993041	1.064617836
0.320220467	2.321364627	1.103060723	1.744465271	0.357544398	1.709691272
1.125072135	1.29050941	1.351465444	1.304859789	0.97680877	0.64021934
0.832602305	1.808131752	1.845473349	1.768633902	0.938160347	0.851278074
0.231378907	2.034209759	2.183880422	2.085699424	0.503604439	0.411343036
0.614717293	3.694432677	4.129932268	3.946643078	0.972279992	0.701168801

Normalized_Patient6_3	Normalized_Patien	Normalized_Patien	Normalized_Patien	Normalized_Patient8_	Normalized_Patient8_
0.583375164	1.323549836	1.318895316	1.330471525	1.606520721	1.671482677
0.485775346	0.691783886	0.679683131	0.733020487	1.35766778	1.401554257
0.235050785	1.677826361	1.579920946	1.672432128	0.653281078	0.688802765
0.374769003	1.424116751	1.487748992	1.531924789	0.732147704	0.793544437
0.232460967	0.423888146	0.390222052	0.532214056	1.104299139	0.98640214
0.227634716	0.487953983	0.288726444	0.304859854	1.007647033	1.085297358
0.284320391	0.269619541	0.282835582	0.323531038	0.975194896	1.06526561
0.288785358	0.349733972	0.301309426	0.35397121	2.006912839	2.076984306
0.327008245	0.352748128	0.333042617	0.358864067	1.037602058	1.040192968
0.591701729	0.311497809	0.296369008	0.300405265	1.523800524	1.637032805
0.541348067	0.461819315	0.413120995	0.449101004	2.087932619	2.184747137
0.91002692	0.792003981	0.745645617	0.710979189	0.888938851	0.794467059
0.835204126	0.608949747	0.57764729	0.628256737	0.897192604	0.976558728
0.547274767	0.452315097	0.40688691	0.416280748	1.048489362	1.154717349
0.59391043	0.701476069	0.650745261	0.667642366	1.279985617	1.352234868
0.346035698	0.38765537	0.477455137	0.458278834	0.994686019	0.942623519
0.732568882	0.624723139	0.563708432	0.645139665	1.116211564	1.191049454
0.462824056	0.250045225	0.252864964	0.303369411	1.601866449	1.636926478
0.88466603	0.529902153	0.489024017	0.478549387	0.979432956	0.98959552
0.713984402	0.602218132	0.55564459	0.628553016	1.168859055	1.269161731
1.131324062	0.953615995	1.181642996	0.906951645	1.07750421	1.135520256
1.430196945	1.602214829	1.973365413	1.894108015	1.035700461	1.50638264
0.581251283	0.832425495	0.901127114	1.153459025	1.757900618	1.958638929
0.78777851	0.635308305	0.577786756	0.612835607	0.949058054	1.018367029
0.396882655	0.807510551	0.782503736	0.812646814	1.12373159	1.172352823
0.703679285	0.740036231	0.700439746	0.727503416	1.15827604	1.207669428

Normalized_Patient8_	Normalized_Patient9	Normalized_Patient9	Normalized_Patient9	Normalized_Patient10_1
1.910936635	0.233005994	0.23159554	0.273940676	1.064163755
1.448251477	0.506105454	0.50724391	0.578855989	0.364161098
0.77893907	0.632062479	0.499086768	0.627851018	2.910124941
0.85712132	0.73178874	0.687417092	0.745200185	2.511773811
1.27938866	0.35437246	0.264582448	0.393169207	0.200748564
0.798921994	0.387770561	0.21433385	0.388740249	0.227165456
1.091088332	0.287423213	0.27298281	0.328475744	0.336474332
2.21951902	0.347786708	0.341677896	0.387432537	0.177235004
1.047689853	0.373327891	0.3352648	0.380867165	0.388964167
1.611345165	0.271605821	0.197281345	0.288139274	0.252930187
2.272275005	0.599409485	0.512689049	0.562243004	0.046981838
0.866267443	0.445698168	0.394554501	0.49307434	0.777249541
1.196511905	0.821760839	0.737778035	0.776943839	0.790084331
1.303380491	0.492144751	0.471962894	0.511956075	0.410753595
1.423664189	0.197114412	0.19763211	0.218239387	0.487424097
1.239876112	0.295868646	0.289959939	0.320565014	0.41991548
1.256599975	0.603900511	0.585250447	0.650630879	0.391137101
1.65394983	0.189105694	0.210821163	0.224719707	0.671272384
1.118446504	0.860132499	0.757157465	0.834144335	0.453117195
1.45525184	0.590764001	0.578103212	0.614934433	0.39937773
1.230406341	0.64925554	0.638098285	0.582887647	0.481459073
1.11723251	0.084480963	0.077459403	0.220719992	1.091978949
1.968533638	0.604085379	0.466474624	0.636668806	0.940529859
1.044340211	0.720802969	0.718333819	0.801702345	0.465503682
1.265724452	0.125368818	0.122034508	0.123105581	1.285392107
1.293442953	0.449419277	0.48494776	0.497505406	0.131919149

Normalized_Patient10_2	Normalized_Patient10_3	Normalized_Patient11_	Normalized_Patient11_	Normalized_Patient11_
1.048725051	1.106065531	0.976291211	0.974255669	1.096551076
0.33964634	0.374727401	2.539501678	2.308914842	2.276493423
2.726844894	2.822294351	0.717923473	0.637768683	0.661254105
2.500895128	2.565492634	0.583985559	0.629350875	0.669839732
0.185511764	0.320375314	3.621657236	3.404663163	3.15084109
0.320157437	0.47555101	3.548576442	3.481825879	3.722232915
0.265996962	0.241373482	4.474514026	4.123251975	3.639424792
0.165358689	0.169326238	2.953693569	2.770404201	2.746731085
0.371584752	0.36935283	3.988899256	3.672380942	3.556721169
0.25305746	0.243614619	3.176014361	3.049645497	2.67233136
0.110020371	0.226222863	2.784823448	2.678209038	2.680416521
0.815025207	0.685254476	1.537487642	1.703665141	1.698162421
0.775281906	0.711988903	0.892373472	0.892852535	0.895898987
0.414106351	0.405336311	1.997204324	1.873357673	2.028771313
0.459377774	0.47594424	2.177958487	2.088988305	1.989379863
0.296539767	0.453860889	1.896499172	1.937248639	2.124355147
0.377859814	0.398772749	1.728612228	1.59413996	1.777482225
0.6116055	0.661267591	3.039789602	2.696750689	2.7824415
0.440839298	0.482707949	1.702725992	1.724828842	1.708354579
0.37985317	0.406511317	1.699248239	1.715380829	2.042409042
0.374371628	0.460816671	1.498192463	1.64606646	1.508558622
1.233573806	0.943448131	1.169473941	1.01458493	0.715034685
0.949766966	1.31097899	0.689120681	0.648631724	0.631528365
0.436843183	0.456498236	1.641084245	1.549537007	1.491486991
1.187121348	1.281472963	0.971233867	0.892562837	0.909914549
0.041079988	0.021383202	1.3376452	1.233674963	1.320355562

Normalized_Patient12_1	Normalized_Patient12_2	Normalized_Patient12_3
2.395757817	2.221647481	2.265975084
2.032197543	1.853940743	1.911235424
3.787428983	3.641634952	3.690219029
3.260904276	3.025489729	3.102442034
1.346991461	1.354411273	1.257631153
1.136410218	1.10130213	1.330937945
1.204722984	1.155085762	1.130417655
1.090388186	0.988866554	1.041504443
1.26448533	1.185597691	1.246176261
1.185880092	1.212584978	1.021527851
1.633839986	1.438437565	1.481304121
0.808308543	0.874363851	0.859421483
1.055210582	1.023335762	1.020096192
1.35801063	1.219593897	1.289344659
1.539010386	1.435128315	1.497272013
1.566296877	1.136590742	1.113955008
1.282513481	1.234120573	1.211950492
1.415482668	1.278659684	1.376993718
1.281762114	1.12879969	1.161076499
1.389449824	1.289567483	1.329292369
1.286703757	1.300204799	0.903214426
1.5782027	1.288146955	1.726666577
1.099470937	1.207287409	1.414149018
1.174443949	1.051008624	1.105975894
1.999717156	1.785042853	1.914541591
0.974476026	0.972822619	1.029377038

Source data of Figure 2F

Target	SLC	SLC2	SLC3	SLC AA	SLC AA2	SLC AA3	CSII	CSII2	CSII3	CSII AA	CSII AA2	CSII AA3	FC	AVE	Normalize	Normalize	Normalize	Normalize	Normalize	Normalize	Normalize	Normalize	Normalize	Normalize	Normalize	Normalize
DHA	0.00406	0.005648	0.004461	0.058088	0.0400133	0.0542542	0.003301	0.002395	0.003542	0.002893	0.00404412	0.00253177	0.11233975	0.01543589	0.262993	0.365892	0.288991	3.763158	2.592226	3.51481	0.21383	0.155188	0.229484	0.187414	0.261994	0.164019
AA	0.002109	0.003377	0.002227	0.028957	0.0228584	0.0260123	0.002739	0.001813	0.002016	0.002474	0.00287815	0.0020152	0.16291001	0.00828966	0.254357	0.407372	0.268661	3.493166	2.757462	3.137923	0.330421	0.21875	0.243188	0.298404	0.347198	0.243098
EPA	0.001209	0.001314	0.002001	0.005521	0.0063293	0.0069733	0.000608	0.001027	0.001948	0.001254	0.00146324	0.00179691	0.34680365	0.00262037	0.461349	0.501324	0.763543	2.107119	2.41544	2.66121	0.232008	0.391886	0.743557	0.478408	0.558408	0.685747
20_3_CE5	0.03285	0.033182	0.030468	0.190686	0.1614514	0.1925423	0.035179	0.030984	0.031694	0.051309	0.0414916	0.03319213	0.3491218	0.07208584	0.455712	0.460311	0.422662	2.645265	2.23971	2.671014	0.488013	0.429822	0.439672	0.711779	0.575586	0.460453
DPA n-3	0.001297	0.001879	0.001021	0.007248	0.0063782	0.0064095	0.000521	0.001584	0.002069	0.002614	0.00145562	0.00243758	0.44080577	0.00290948	0.445837	0.645719	0.350784	2.491151	2.192199	2.202982	0.17909	0.54431	0.711275	0.898545	0.500302	0.837805
rLN_2	0.000432	8.76E-05	8.34E-05	0.000406	0.0003637	0	8.68E-05	8.56E-05	8.86E-05	9.64E-05	0.00025825	8.1685E-05	0.50810058	0.00017248	2.503274	0.507744	0.483338	2.353834	2.108839	0	0.50344	0.496081	0.513506	0.559131	1.497231	0.473582
DPA n-6	0.001014	0.000992	0.000671	0.001493	0.0021877	0.0029472	0.001058	0.000725	0.000948	0.000893	0.00087841	0.00023299	0.50882516	0.00116994	0.867131	0.847668	0.573237	1.276086	1.869972	2.519115	0.904333	0.619585	0.809942	0.76296	0.750821	0.19915
20_2_CE5	0.014766	0.009677	0.00796	0.030865	0.0243231	0.0270374	0.008726	0.008342	0.009168	0.020106	0.00886555	0.01041366	0.57247278	0.01502074	0.983011	0.644225	0.529956	2.054793	1.619303	1.800006	0.580919	0.555397	0.610342	1.338544	0.59022	0.693285
rLN_1	0.000173	0	8.34E-05	0.000162	0.0002909	0.000168	0.000174	8.56E-05	8.86E-05	9.64E-05	0.00017214	0.00016337	0.88872412	0.00013809	1.250461	0	0.603697	1.17599	2.106857	1.216491	1.257609	0.619613	0.641377	0.698363	1.24652	1.183023
16_1_CE5	0.072392	0.082314	0.067014	0.141194	0.1338438	0.1505382	0.076848	0.082694	0.07244	0.123625	0.12092962	0.1085223	0.90384938	0.10269614	0.704914	0.801526	0.652549	1.374871	1.303299	1.46586	0.748302	0.805228	0.705381	1.20379	1.177548	1.056732
20_1_CE5	0.02961	0.034945	0.034181	0.051226	0.0393919	0.0393132	0.03404	0.036022	0.029938	0.03704	0.03873424	0.03388986	0.91689357	0.03652763	0.810622	0.956679	0.935759	1.402395	1.078414	1.076258	0.93189	0.986155	0.819595	1.014035	1.060409	0.927787
15_1_CE5	0.020809	0.023032	0.017838	0.027942	0.0153862	0.0252845	0.017844	0.012234	0.011424	0.025652	0.03340336	0.02001246	0.92538307	0.02090509	0.995392	1.101757	0.853297	1.336591	0.736	1.209489	0.853558	0.585236	0.546467	1.227054	1.597858	0.957301
18_3_CE5	0.08177	0.07697	0.075203	0.086649	0.0845317	0.0966171	0.07494	0.063482	0.078087	0.089879	0.08161765	0.08183404	0.93641972	0.08096517	1.009944	0.95066	0.928837	1.070195	1.044051	1.193317	0.925588	0.784067	0.964452	1.110099	1.008059	1.010731
13_0_CE5	0.061433	0.058162	0.046821	0.104384	0.0466933	0.0555356	0.059576	0.051814	0.054337	0.071286	0.05680147	0.05848492	0.94442723	0.06044403	1.016363	0.962244	0.774617	1.72696	0.772505	0.918794	0.985642	0.857225	0.898958	1.179367	0.939737	0.967588
18_2_CE5	1.399631	1.182741	1.333927	1.356701	1.4278739	1.3846745	1.149669	1.224223	1.475007	1.36246	1.42909664	1.33491154	0.98637306	1.33840957	1.045742	0.883691	0.996651	1.013666	1.066844	1.034567	0.858981	0.914685	1.102059	1.017969	1.067757	0.997386
18_1_CE5	3.832982	4.434945	3.738555	4.188754	3.4110075	4.2362891	4.100662	3.844949	3.812483	4.566049	3.65283613	3.63568403	0.99035883	3.95459974	0.969247	1.121465	0.945369	1.059211	0.862542	1.071231	1.036935	0.972273	0.964063	1.154617	0.923693	0.919356
17_0_CE5	0.391728	0.407694	0.373856	0.43349	0.3610741	0.4131215	0.386755	0.370138	0.367468	0.453957	0.42909664	0.40617992	1.01370508	0.39954659	0.980432	1.020393	0.9357	1.084955	0.90371	1.033976	0.967985	0.926396	0.919713	1.136181	1.073959	1.016602
18_0_CE5	7.123288	7.790542	7.222787	7.723557	6.6200621	7.6832394	7.668874	7.392326	7.354769	8.811415	7.56302521	7.11687017	1.03948519	7.50589629	0.949026	1.037923	0.962282	1.028999	0.881982	1.023627	1.021713	0.984869	0.979866	1.173932	1.007611	0.948171
20_0_CE5	0.08127	0.110152	0.088352	0.111964	0.0909898	0.114736	0.108636	0.086583	0.091705	0.137511	0.09847689	0.10822327	1.05635629	0.10238327	0.79378	1.075882	0.862953	1.09358	0.888717	1.120652	1.061069	0.845677	0.895702	1.343101	0.961846	1.057041
15_0_CE5	0.364067	0.341972	0.352238	0.325242	0.3067022	0.3710917	0.315497	0.318716	0.320184	0.42689	0.40651261	0.38998256	1.05650234	0.35325785	1.0306	0.968051	0.997113	0.920692	0.868211	1.050484	0.893106	0.902218	0.906374	1.208438	1.150753	1.10396
16_0_CE5	9.149104	9.529789	8.738555	9.348526	7.900577	9.4028703	9.75894	9.274341	9.519049	10.88555	9.31460084	9.0605532	1.06923722	9.32353842	0.981291	1.022121	0.937257	1.00268	0.84738	1.008509	1.046699	0.994723	1.02097	1.167535	0.999041	0.971793
14_1_CE5	0.006994	0.004291	0.005837	0.008932	0.0106214	0.010164	0.008596	0.005646	0.004429	0.013019	0.01282563	0.00612509	1.08113835	0.00812327	0.861008	0.528196	0.718519	1.099603	1.307527	1.251222	1.058198	0.695046	0.545166	1.602622	1.578875	0.754018
12_0_CE5	0.491043	0.489981	0.465412	0.491206	0.4695961	0.5171707	0.478411	0.485252	0.462037	0.634893	0.59401261	0.54622477	1.09452169	0.51043662	0.962006	0.959926	0.911792	0.962326	0.919989	1.013193	0.937258	0.95066	0.905181	1.243823	1.163734	1.070113
20_5_CE5	0.185643	0.182714	0.194634	0.200669	0.1857301	0.21143	0.17804	0.162334	0.179465	0.270197	0.24264706	0.24231248	1.09835725	0.20298458	0.914566	0.900139	0.95886	0.988591	0.914996	1.041606	0.87711	0.799734	0.884131	1.331121	1.195397	1.193748
aLN_rLN	0.001079	0.000963	0.00075	0.001583	0.0003637	0.001092	0.001129	0.001326	0.001417	0.001061	0.00176471	0.00057189	1.24651183	0.00109181	0.988534	0.882384	0.687164	1.450207	0.33315	1.000175	1.033824	1.214756	1.298011	0.971687	1.616308	0.5238
14_0_CE5	0.689937	0.855464	0.658698	0.874907	0.6624501	0.8546899	0.858543	0.813104	0.734396	1.391292	1.05488445	0.99077997	1.27128244	0.86992867	0.793096	0.983372	0.757186	1.005723	0.761499	0.982483	0.986912	0.934679	0.844203	1.599317	1.21261	1.138921

Patient information described in Figure 3E

number	Information	FATP2	OS_Dead1 Alive0	OS_months	PFS_months	PFS_Progression1	Age	sex	Hans_01v 2009	BCN_Nodal_vs_Extranodal_DLBCL 2	LDH	B2M	ECOG_PS	Performance_Status 01	chmo	treatment	respons	sIL2R	B_symptoms sato	CSAAS	Stage_III_IV	IPI	IPI	IPI_L_vs_H	CD3	CD5	CD20	CD10	GCET1	MUM1	BCL2	BCL6	FOXP1	FISH_BCL2	FISH_CMYC	FISH_doubleHit	sIL2Rb
#1	TMA30_DLBCL	+	0	176	115	0	31	1	0	1	345	1	NA	0	RCHOP8	1	CR	394	A	2A	0	L	0	0	0	0	1	1	0	0	0	0	1	0	0	0	1
#2	TMA30_DLBCL	+	1	9	8	1	48	0	1	3	411	2	NA	NA	RCHOP8	1	PR	822	A	2A	0	L	0	0	0	0	1	0	1	0	1	0	1	0	0	0	1
#3	TMA30_DLBCL	-	1	104	90	1	68	1	1	0	179	NA	NA	NA	RCHOP6	1	CR	367	A	3A	1	L	0	0	0	0	1	0	0	1	1	1	1	0	0	0	1
#4	TMA30_DLBCL	+	0	134	100	0	73	0	1	0	185	NA	NA	0	RCHOP6	1	CR	3160	B	3B	1	LI	1	0	0	0	1	0	0	0	1	0	0	NA	NA	NA	3
#5	TMA30_DLBCL	+	0	33	29	0	60	1	1	0	318	2	NA	NA	R8CHOP1	1	CR	1020	A	4A	1	NA	NA	NA	0	0	1	0	0	1	1	0	1	0	0	0	2
#6	TMA30_DLBCL	-	0	112	109	0	39	0	1	0	286	NA	NA	NA	RCHOP8	1	CR	635	A	4A bulky	1	NA	NA	NA	0	0	1	0	1	0	1	0	0	NA	NA	NA	1
#7	TMA30_DLBCL	+	0	11	7	1	53	1	0	3	165	NA	NA	0	RCHOP8	1	PD	540	NA	1E	0	L	0	0	0	0	1	1	0	1	0	1	1	0	0	0	1
#8	TMA30_DLBCL	+	0	103	103	0	84	1	1	3	540	NA	NA	NA	RCHOP6	1	CR	6180	NA	2	0	NA	NA	NA	0	0	1	0	0	1	1	0	1	0	0	0	3
#9	TMA30_DLBCL	-	0	74	74	0	53	0	1	0	422	3	NA	NA	RCHOP8	1	CR	6870	NA	NA	NA	NA	NA	NA	0	0	1	0	0	1	0	0	1	0	0	0	3
#10	TMA30_DLBCL	+	0	74	74	0	75	1	1	0	252	NA	NA	NA	RCHOP6	1	CR	704	A	2A	0	L	0	0	0	0	0	0	0	1	1	0	1	0	0	0	1
#11	TMA30_DLBCL	+	1	110	97	0	71	1	0	0	316	NA	NA	NA	RCHOP6	1	CR	1160	B	2B	0	LI	1	0	0	0	1	1	1	0	1	1	1	0	0	0	2
#12	TMA30_DLBCL	+	0	151	146	0	69	1	1	3	277	2	NA	0	6+MTX IT2	2	CR	1030	A	2A	0	LI	1	0	0	0	1	0	1	0	1	0	1	0	0	0	2
#13	TMA30_DLBCL	-	1	8	7	1	71	0	1	0	316	3	NA	NA	RCHOP1	1	PR	7350	A	3A	1	LI	1	0	0	0	1	0	0	0	1	0	1	0	0	0	3
#14	TMA30_DLBCL	-	1	37	25	1	73	0	1	0	253	NA	NA	NA	RCHOP6	1	CR	1090	A	2A	0	NA	NA	NA	0	0	1	0	0	1	1	1	1	0	0	0	2
#15	TMA30_DLBCL	+	0	82	82	0	60	0	0	3	1280	NA	NA	NA	RCHOP5	1	CR	1250	A	2A	0	LI	1	0	0	0	1	1	1	1	1	1	1	1	0	0	2
#16	TMA30_DLBCL	-	1	2	2	1	68	1	0	0	207	2	NA	NA	RCHOP2	1	NA	443	A	3A	1	NA	NA	NA	0	0	1	0	1	0	1	1	1	0	0	0	1
#17	TMA30_DLBCL	-	0	81	81	0	28	1	0	1	149	2	NA	0	RCHOP6	1	CR	477	A	1A	0	L	0	0	0	0	1	1	0	1	1	1	1	0	0	0	1
#18	TMA30_DLBCL	+	0	141	79	0	65	1	1	1	164	1	NA	0	RCHOP6	1	CR	407	NA	1A	0	LI	1	0	0	0	1	0	0	0	0	0	1	0	0	0	1
#19	TMA30_DLBCL	-	1	71	4	1	74	1	1	3	256	4	NA	0	OPE	3	NA	1650	NA	NA	NA	NA	NA	NA	0	0	1	0	NA	1	1	1	NA	0	0	0	2
#20	TMA30_DLBCL	+	0	110	76	0	63	1	1	2	144	2	NA	0	HOP2+R6	2	CR	677	NA	1 A	0	L	0	0	NA	NA	NA	0	NA	1	NA	1	NA	0	0	0	1
#21	TMA30_DLBCL	-	0	134	7	1	34	1	NA	0	243	1	NA	0	CHOP8	2	PR	2230	NA	2A	0	L	0	0	NA	NA	NA	NA	NA	NA	NA	NA	NA	0	0	0	2
#22	TMA30_DLBCL	-	1	7	5	1	82	0	1	2	358	NA	NA	0	NA	NA	PD	13600	NA	4A	1	NA	NA	NA	NA	NA	NA	NA	NA	NA	NA	NA	NA	0	0	0	3
#23	TMA30_DLBCL	+	0	122	70	0	14	1	0	0	284	1	NA	0	-ルGroupII	3	NA	696	NA	1	0	NA	NA	NA	NA	NA	NA	NA	NA	NA	NA	NA	NA	0	0	0	1
#24	TMA30_DLBCL	+	1	6	3	1	69	1	1	3	317	NA	NA	0	+RTx50Gy	3	PD	780	NA	NA	NA	NA	NA	NA	0	0	1	0	NA	1	1	1	NA	0	0	0	1
#25	TMA30_DLBCL	-	1	16	11	1	56	0	1	3	1196	3	NA	0	RCHOP8	1	CR	3150	A	4A	1	HI	2	1	0	1	1	0	NA	1	1	1	NA	0	0	0	3
#26	TMA30_DLBCL	+	0	125	64	0	69	0	1	0	339	2	NA	0	RCHOP8	1	CR	1190	B	2B	0	LI	1	0	0	0	1	0	NA	1	1	1	NA	0	0	0	2
#27	TMA30_DLBCL	-	1	19	19	1	83	0	1	1	351	NA	NA	0	ɔTx39.6Gy	2	CR	1980	A	4A	0	LI	1	0	0	0	1	0	NA	1	1	1	NA	0	0	0	2
#28	TMA30_DLBCL	+	1	13	10	1	67	1	1	3	644	3	NA	1	CP+ETP3	2	CR	NA	A	4A	1	H	3	1	0	0	1	0	NA	1	1	1	NA	1	1	1	NA
#29	TMA30_DLBCL	+	1	5	NA	NA	97	0	1	3	NA	NA	NA	NA	NA	NA	NA	NA	NA	NA	NA	NA	NA	NA	0	0	1	0	NA	1	1	0	NA	0	0	0	NA
#30	TMA30_DLBCL	+	0	119	57	0	69	0	1	0	727	3	NA	0	RCHOP8	1	CR	4940	A	3A	1	HI	2	1	0	0	1	0	NA	1	1	1	NA	0	0	0	3
#31	07-1	+	0	66	NA	NA	69	0	NA	NA	543	NA	NA	0	NA	NA	NA	7130	NA	NA	3	NA	NA	NA	NA	NA	NA	NA	NA	NA	NA	NA	NA	NA	NA	NA	NA
#32	07-10	-	0	57	NA	NA	77	1	NA	NA	159	NA	NA	0	NA	NA	L	679	NA	NA	1	NA	NA	NA	NA	NA	NA	NA	NA	NA	NA	NA	NA	NA	NA	NA	NA
#33	07-11	+	0	56	NA	NA	51	1	NA	NA	236	NA	NA	0	NA	NA	L	368	NA	NA	1	NA	NA	NA	NA	NA	NA	NA	NA	NA	NA	NA	NA	NA	NA	NA	NA
#34	07-2	-	1	4	NA	NA	73	1	NA	NA	372	NA	NA	1	NA	NA	LI	2970	NA	NA	2	NA	NA	NA	NA	NA	NA	NA	NA	NA	NA	NA	NA	NA	NA	NA	NA
#35	07-3	-	1	4	NA	NA	65	1	NA	NA	1284	NA	NA	0	NA	NA	HI	6710	NA	NA	3	NA	NA	NA	NA	NA	NA	NA	NA	NA	NA	NA	NA	NA	NA	NA	NA
#36	07-4	-	1	8	NA	NA	85	0	NA	NA	268	NA	NA	1	NA	NA	LI	902	NA	NA	1	NA	NA	NA	NA	NA	NA	NA	NA	NA	NA	NA	NA	NA	NA	NA	NA
#37	07-5	-	0	61	NA	NA	52	0	NA	NA	171	NA	NA	0	NA	NA	L	422	NA	NA	1	NA	NA	NA	NA	NA	NA	NA	NA	NA	NA	NA	NA	NA	NA	NA	NA
#38	07-6	-	1	17	NA	NA	65	1	NA	NA	879	NA	NA	NA	NA	NA	NA	6690	NA	NA	4	NA	NA	NA	NA	NA	NA	NA	NA	NA	NA	NA	NA	NA	NA	NA	NA
#39	07-7	+	0	57	NA	NA	77	0	NA	NA	206	NA	NA	0	NA	NA	L	546	NA	NA	1	NA	NA	NA	NA	NA	NA	NA	NA	NA	NA	NA	NA	NA	NA	NA	NA
#40	07-8	-	0	56	NA	NA	63	1	NA	NA	142	NA	NA	0	NA	NA	L	677	NA	NA	1	NA	NA	NA	NA	NA	NA	NA	NA	NA	NA	NA	NA	NA	NA	NA	NA
#41	07-9	-	1	13	NA	NA	76	1	NA	NA	1098	NA	NA	1	NA	NA	LI	4350	NA	NA	2	NA	NA	NA	NA	NA	NA	NA	NA	NA	NA	NA	NA	NA	NA	NA	NA
#42	09-1	+	0	38	NA	NA	37	1	NA	NA	168	NA	NA	0	NA	NA	L	499	NA	NA	1	NA	NA	NA	NA	NA	NA	NA	NA	NA	NA	NA	NA	NA	NA	NA	NA
#43	09-10	+	0	30	NA	NA	77	0	NA	NA	291	NA	NA	1	NA	NA	LI	1120	NA	NA	2	NA	NA	NA	NA	NA	NA	NA	NA	NA	NA	NA	NA	NA	NA	NA	NA
#44	09-11	+	0	30	NA	NA	57	1	NA	NA	161	NA	NA	0	NA	NA	L	241	NA	NA	1	NA	NA	NA	NA	NA	NA	NA	NA	NA	NA	NA	NA	NA	NA	NA	NA
#45	09-2	-	1	5	NA	NA	62	0	NA	NA	163	NA	NA	3	NA	NA	H	985	NA	NA	4	NA	NA	NA	NA	NA	NA	NA	NA	NA	NA	NA	NA	NA	NA	NA	NA
#46	09-3	-	1	38	NA	NA	72	0	NA	NA	910	NA	NA	NA	NA	NA	HI	NA	NA	NA	4	NA	NA	NA	NA	NA	NA	NA	NA	NA	NA	NA	NA	NA	NA	NA	NA
#47	09-4	+	0	36	NA	NA	55	1	NA	NA	366	NA	NA	0	NA	NA	H	1370	NA	NA	3	NA	NA	NA	NA	NA	NA	NA	NA	NA	NA	NA	NA	NA	NA	NA	NA
#48	09-5	+	0	34	NA	NA	65	1	NA	NA	151	NA	NA	0	NA	NA	HI	491	NA	NA	3	NA	NA	NA	NA	NA	NA	NA	NA	NA	NA	NA	NA	NA	NA	NA	NA
#49	09-6	+	0	33	NA	NA	66	1	NA	NA	173	NA	NA	0	NA	NA	LI	880	NA	NA	2	NA	NA	NA	NA	NA	NA	NA	NA	NA	NA	NA	NA	NA	NA	NA	NA
#50	09-7	+	0	32	NA	NA	62	1	NA	NA	186	NA	NA	0	NA	NA	L	605	NA	NA	1	NA	NA	NA	NA	NA	NA	NA	NA	NA	NA	NA	NA	NA	NA	NA	NA
#51	09-8	-	0	30	NA	NA	56	1	NA	NA	167	NA	NA	0	NA	NA	L	240	NA	NA	1	NA	NA	NA	NA	NA	NA	NA	NA	NA	NA	NA	NA	NA	NA	NA	NA
#52	09-9	-	1	11	NA	NA	38	0	NA	NA	740	NA	NA	3	NA	NA	H	16400	NA	NA	4	NA	NA	NA	NA	NA	NA	NA	NA	NA	NA	NA	NA	NA	NA	NA	NA
#53	10-1	-	0	28	NA	NA	78	1	NA	NA	647	NA	NA	4	NA	NA	H	7280	NA	NA	4	NA	NA	NA	NA	NA	NA	NA	NA	NA	NA	NA	NA	NA	NA	NA	NA
#54	10-10	+	0	20	NA	NA	76	1	NA	NA	470	NA	NA	1	NA	NA	L	1050	NA	NA	2	NA	NA	NA	NA	NA	NA	NA	NA	NA	NA	NA	NA	NA	NA	NA	NA
#55	10-11	-	0	8	NA	NA	61	0	NA	NA	195	NA	NA	0	NA	NA	LI	551	NA	NA	2	NA	NA	NA	NA	NA	NA	NA	NA	NA	NA	NA	NA	NA	NA	NA	NA
#56	10-12	+	0	26	NA	NA	69	0	NA	NA	890	NA	NA	0	NA	NA	H	10400	NA	NA	4	NA	NA	NA	NA	NA	NA	NA	NA	NA	NA	NA	NA	NA	NA	NA	NA
#57	10-13	+	0	19	NA	NA	50	0	NA	NA	1185	NA	NA	2	NA	NA	HI	2460	NA	NA	3	NA	NA	NA	NA	NA	NA	NA	NA	NA	NA	NA	NA	NA	NA	NA	NA
#58	10-2	-	0	27	NA	NA	71	0	NA	NA	167	NA	NA	0	NA	NA	L	557	NA	NA	1	NA	NA	NA	NA	NA	NA	NA	NA	NA	NA	NA	NA	NA	NA	NA	NA
#59	10-3	+	0	27	NA	NA	50	0	NA	NA	150	NA	NA	0	NA	NA	L	236	NA	NA	1	NA	NA	NA	NA	NA	NA	NA	NA	NA	NA	NA	NA	NA	NA	NA	NA
#60	10-4	+	0	26	NA	NA	34	1	NA	NA	137	NA	NA	0	NA	NA	L	503	NA	NA	2	NA	NA	NA	NA	NA	NA	NA	NA	NA	NA	NA	NA	NA	NA	NA	NA
#61	10-5	-	1	12	NA	NA	84	1	NA	NA	220	NA	NA	1	NA	NA	LI	787	NA	NA	1	NA	NA	NA	NA	NA	NA	NA	NA	NA	NA	NA	NA	NA	NA	NA	NA
#62	10-6	+	0	23	NA	NA	72	1	NA	NA	214	NA	NA	0	NA	NA	L	1930	NA	NA	2	NA	NA	NA	NA	NA	NA	NA	NA	NA	NA	NA	NA	NA	NA	NA	NA
#63	10-7	-	0	12	NA	NA	78	1	NA	NA	232	NA	NA	0	NA	NA	L	622	NA	NA	2	NA	NA	NA	NA	NA	NA	NA	NA	NA	NA	NA	NA	NA	NA	NA	NA
#64	10-8	-	0	23	NA	NA	80	0	NA	NA	206	NA	NA	0	NA	NA	L	946	NA	NA	2	NA	NA	NA	NA	NA	NA	NA	NA	NA	NA	NA	NA	NA	NA	NA	NA
#65	10-9	-	1	37	NA	NA	75	0	NA	NA	300	NA	NA	0	NA	NA	HI	1060	NA	NA	3	NA	NA	NA	NA	NA	NA	NA	NA	NA	NA	NA	NA	NA	NA	NA	NA
#66	11-1	+	0	14	NA	NA	67	0	NA	NA	182	NA	NA	0	NA	NA	L	407	NA	NA	2	NA	NA	NA	NA	NA	NA	NA	NA	NA	NA	NA	NA	NA	NA	NA	NA
#67	11-10	+	0	11	NA	NA	70	0	NA	NA	434	NA	NA	1	NA	NA	HI	3060	NA	NA	4	NA	NA	NA	NA	NA	NA	NA	NA	NA	NA	NA	NA	NA	NA	NA	NA
#68	11-11	-	1	9	NA	NA	76	0	NA	NA	190	NA	NA	0	NA	NA	HI	4690	NA	NA	4	NA	NA	NA	NA	NA	NA	NA	NA	NA	NA	NA	NA	NA	NA	NA	NA
#69	11-12	+	0	10	NA	NA	78	0	NA	NA	542	NA	NA	0	NA	NA	H	1410	NA	NA	3	NA	NA	NA	NA	NA	NA	NA	NA	NA	NA	NA	NA	NA	NA	NA	NA
#70	11-13	+	0	10	NA	NA	61	0	NA	NA	1236	NA	NA	>2	NA	NA	H	1520	NA	NA	4	NA	NA	NA	NA	NA	NA	NA	NA	NA	NA	NA	NA	NA	NA	NA	NA
#71	11-14	-	0	9	NA	NA	80	0	NA	NA	308	NA	NA	0	NA	NA	HI	NA	NA	NA	3	NA	NA	NA	NA	NA	NA	NA	NA	NA	NA	NA	NA	NA	NA	NA	NA
#72	11-15	-	1	18	NA	NA	59	1	NA	NA	445	NA	NA	0	NA	NA	HI	10800	NA	NA	3	NA	NA	NA	NA	NA	NA	NA	NA	NA	NA	NA	NA	NA	NA	NA	NA
#73	11-16	+	0	7	NA	NA	68	1	NA	NA	294	NA	NA	0	NA	NA	HI	597	NA	NA	4	NA	NA	NA	NA	NA	NA	NA	NA	NA	NA	NA	NA	NA	NA	NA	NA
#74	11-17	+	0	7	NA	NA	41	0	NA	NA	143	NA	NA	0	NA	NA	L	330	NA	NA	2	NA	NA	NA	NA	NA	NA	NA	NA	NA	NA	NA	NA	NA	NA	NA	NA
#75	11-18	-	1	2	NA	NA	81	1	NA	NA	334	NA	NA	3	NA	NA	H	1560	NA	NA	4	NA	NA	NA	NA	NA	NA	NA	NA	NA	NA	NA	NA	NA	NA	NA	NA
#76	11-19	-	0	12	NA	NA	69	0	NA	NA	276	NA	NA	0	NA	NA	LI	482	NA	NA	1	NA	NA	NA	NA	NA	NA	NA	NA	NA	NA	NA	NA	NA	NA	NA	NA
#77	11-2	+	0	14	NA	NA	76	1	NA	NA	192	NA	NA	0	NA	NA	LI	1070	NA	NA	3	NA	NA	NA	NA	NA	NA	NA	NA	NA	NA	NA	NA	NA	NA	NA	NA
#78	11-20	-	1	4	NA	NA	76	1	NA	NA	484	NA	NA	3	NA	NA	H	3530	NA	NA	3	NA	NA	NA	NA	NA	NA	NA	NA	NA	NA	NA	NA	NA	NA	NA	NA
#79	11-21	+	0	5	NA	NA	43	0	NA	NA	442	NA	NA	0	NA	NA	LI	4020	NA	NA	3	NA	NA	NA	NA	NA	NA	NA	NA	NA	NA	NA	NA	NA	NA	NA	NA
#80	11-3	+	0	15	NA	NA	72	1	NA	NA	787	NA	NA	0	NA	NA	L	4500	NA	NA	2	NA	NA	NA	NA	NA	NA	NA	NA	NA	NA	NA	NA	NA	NA	NA	NA
#81	11-4	+	0	14	NA	NA	58	0	NA	NA	199	NA	NA	0	NA	NA	LI	308	NA	NA	3	NA	NA	NA	NA	NA	NA	NA	NA	NA	NA	NA	NA	NA	NA	NA	NA
#82	11-5	-	0	13	NA	NA	43	1	NA	NA	186	NA	NA	0	NA	NA	HI	287	NA	NA	4	NA	NA	NA	NA	NA	NA	NA	NA	NA	NA	NA	NA	NA	NA	NA	NA
#83	11-6	+	0	26	NA	NA	62	0	NA	NA	193	NA	NA	0	NA	NA	LI	772	NA	NA	4	NA	NA	NA	NA	NA	NA	NA	NA	NA	NA	NA	NA	NA	NA	NA	NA
#84	11-7	+	0	12	NA	NA	57	0	NA	NA	173	NA	NA	0	NA	NA	L	323	NA	NA	1	NA	NA	NA	NA	NA	NA	NA	NA	NA	NA	NA	NA	NA	NA	NA	NA
#85	11-8	+	0	12	NA	NA	48	1	NA	NA	404	NA	NA	0	NA	NA	HI	2580	NA	NA	4	NA	NA	NA	NA	NA	NA	NA	NA	NA	NA	NA	NA	NA	NA	NA	NA
#86	11-9	-	0	7	NA	NA	64	1	NA	NA	157	NA	NA	0	NA	NA	L	768	NA	NA	1	NA	NA	NA	NA	NA	NA	NA	NA	NA	NA	NA	NA	NA	NA	NA	NA

number	Information	FATP2	OS_Dead1_Alive0	OS_months	PFS_months	PFS_Progression1	Age	sex	Hans_01v2009	BCN_Nodal_vs_Extranodal_DLBCL_2	LDH	B2M	ECOG_PS	Performance_Status_01	chmo	treatment	respons	sIL2R	B_symptoms_sato	CSAAS	Stage_III_IV	IPI	IPI_	IPI_L_vs_H	CD3	CD5	CD20	CD10	GCET1	MUM1	BCL2	BCL6	FOXP1	FISH_BCL2	FISH_CMYC	FISH_doubleHit	sIL2Rb
#1	15-P17-nonT/9-TR*-T2.3	-	1	0.10	NA	NA	63	NA	NA	NA	1798	NA	NA	NA	NA	NA	NA	9710	NA	NA	NA	NA	NA	NA	NA	NA	NA	NA	NA	NA	NA	NA	NA	NA	NA	NA	NA
#2	TMA_DLBCL_EBER+Rituximab+	-	0	100.03	NA	NA	64	NA	NA	NA	209	NA	NA	NA	NA	NA	NA	1000	NA	NA	NA	NA	NA	NA	NA	NA	NA	NA	NA	NA	NA	NA	NA	NA	NA	NA	NA
#3	TMA_DLBCL_EBER+Rituximab+	+	1	28.26	NA	NA	80	NA	NA	NA	293	NA	NA	NA	NA	NA	NA	1990	NA	NA	NA	NA	NA	NA	NA	NA	NA	NA	NA	NA	NA	NA	NA	NA	NA	NA	NA
#4	TMA_DLBCL_EBER+Rituximab+	-	1	3.00	NA	NA	69	NA	NA	NA	219	NA	NA	NA	NA	NA	NA	6780	NA	NA	NA	NA	NA	NA	NA	NA	NA	NA	NA	NA	NA	NA	NA	NA	NA	NA	NA
#5	TMA_DLBCL_EBER+Rituximab+	-	0	0.35	NA	NA	75	NA	NA	NA	210	NA	NA	NA	NA	NA	NA	NA	NA	NA	NA	NA	NA	NA	NA	NA	NA	NA	NA	NA	NA	NA	NA	NA	NA	NA	NA
#6	TMA_DLBCL_EBER+Rituximab+	-	1	9.87	NA	NA	78	NA	NA	NA	249	NA	NA	NA	NA	NA	NA	374	NA	NA	NA	NA	NA	NA	NA	NA	NA	NA	NA	NA	NA	NA	NA	NA	NA	NA	NA
#7	TMA_DLBCL_EBER+Rituximab+	+	0	107.81	NA	NA	59	NA	NA	NA	154	NA	NA	NA	NA	NA	NA	1250	NA	NA	NA	NA	NA	NA	NA	NA	NA	NA	NA	NA	NA	NA	NA	NA	NA	NA	NA
#8	TMA_DLBCL_EBER+Rituximab+	-	1	1.45	NA	NA	69	NA	NA	NA	363	NA	NA	NA	NA	NA	NA	1390	NA	NA	NA	NA	NA	NA	NA	NA	NA	NA	NA	NA	NA	NA	NA	NA	NA	NA	NA
#9	TMA_DLBCL_EBER+Rituximab-	+	0	50.83	NA	NA	NA	NA	NA	NA	NA	NA	NA	NA	NA	NA	NA	NA	NA	NA	NA	NA	NA	NA	NA	NA	NA	NA	NA	NA	NA	NA	NA	NA	NA	NA	NA
#10	TMA_DLBCL_EBER+Rituximab-	+	0	83.73	NA	NA	NA	NA	NA	NA	NA	NA	NA	NA	NA	NA	NA	NA	NA	NA	NA	NA	NA	NA	NA	NA	NA	NA	NA	NA	NA	NA	NA	NA	NA	NA	NA
#11	TMA_DLBCL_EBER+Rituximab-	+	0	9.73	NA	NA	NA	NA	NA	NA	NA	NA	NA	NA	NA	NA	NA	NA	NA	NA	NA	NA	NA	NA	NA	NA	NA	NA	NA	NA	NA	NA	NA	NA	NA	NA	NA
#12	TMA_DLBCL_EBER+Rituximab-	+	0	93.53	NA	NA	NA	NA	NA	NA	NA	NA	NA	NA	NA	NA	NA	NA	NA	NA	NA	NA	NA	NA	NA	NA	NA	NA	NA	NA	NA	NA	NA	NA	NA	NA	NA
#13	TMA_DLBCL_EBER+Rituximab-	+	1	20.57	NA	NA	NA	NA	NA	NA	NA	NA	NA	NA	NA	NA	NA	NA	NA	NA	NA	NA	NA	NA	NA	NA	NA	NA	NA	NA	NA	NA	NA	NA	NA	NA	NA
