# Supplementary material for: Unique lipid composition maintained by extracellular blockade leads to prooncogenicity
Source: Cell Death Discov. 2024 May 8;10:221. doi: 10.1038/s41420-024-01971-y (PMC11079073; doi:10.1038/s41420-024-01971-y)
Supplement: Supplementary file 3 — Supplementary Data Table 1 [file 41420_2024_1971_MOESM3_ESM.docx]

**Supplemental Data Table 1. Primers used in this study.**

| primers | |
| --- | --- |
| *FADS1* | Forward: 5’- GATGCCTCGACACAATTACC-3’ |
|  | Reverse: 5’- CTGCCCTGACTCCTTTAGTG-3’ |
| *FADS2* | Forward: 5’- ACAAGGATCCCGATGTGAAC-3’ |
|  | Reverse: 5’- TTCGTGCTGGTGATTGTAGG-3’ |
| *SLC27A1* | Forward: 5’- CTGCCCTTAAATGAGGCAGTCT-3’ |
|  | Reverse: 5’- AACAGCTTCAGAGGGCGAAG-3’ |
| *SLC27A2* | Forward: 5’- TCTTGGATGACACAGCAAAAATGT-3’ |
|  | Reverse: 5’- TCAGAGTTTCAGGGTTTTAGCACTT-3’ |
| *SLC27A3* | Forward: 5’- TACCTGCCCCTCACAACTGC-3’ |
|  | Reverse: 5’- GTGGAAGTTCTCAGATTCGAAGG-3’ |
| *SLC27A4* | Forward: 5’- TTCTGTGAAAGTCTCATGTCCAAGT-3’ |
|  | Reverse: 5’- TCTCAGCCTGGGAACCAGAG-3’ |
| *SLC27A6* | Forward: 5’- CTTCGTTCACGTGTGGTTCG-3’ |
|  | Reverse: 5’- AATGTTGGTGTTGAGAAAGGCC-3’ |
| *GAPDH* | Forward: 5’-CTGCACCACCAACTGCTTAG-3’ |
|  | Reverse: 5’-TTCAGCTCAGGGATGACCTTG-3’ |
